# Speed Control of Single Molecules on a Surface

**DOI:** 10.1002/anie.5538086

**Published:** 2026-09-08

**Authors:** Donato Civita, Matthew J. Timm, Julia Lanz, Christophe Nacci, Stefan Hecht, Leonhard Grill

**Affiliations:** ^1^ Department of Physical Chemistry University of Graz Graz Austria; ^2^ Department of Chemistry and Center for the Science of Materials Berlin Humboldt‐Universität Zu Berlin Berlin Germany

**Keywords:** atomic defect, molecular dynamics, scanning probe microscopy, single‐molecule manipulation, surface chemistry

## Abstract

Laws of motion are of fundamental importance for the understanding of dynamic systems. At the atomic scale, the kinetic energy of molecules controls chemical reactions. The velocity of individual molecules, however, has never been measured, because it is difficult to determine time–distance relationships. Here, we show how the real speed of a single molecule moving across a Ag(111) surface is measured at the microsecond time scale. We found that atomic defects extend the travelling time by orders of magnitude, even at distances of few nanometers from the molecule and with high sensitivity to their precise location. Statistical measurements, obtained by repetitive translation of a single molecule in an oscillating electric field, give insight into the time scales of molecular motion in a confined environment. The strong influence of the defects underscores the need for their investigation during chemical reactions or collision experiments at variable velocities and geometries on a surface.

## Graphical abstract

The speed of a single molecule moving across a silver crystal surface is determined with the tip of a scanning tunneling microscope (STM). Travelling distances Δ*x* and travelling times Δ*t*, which are determined simultaneously with high precision, allow to directly measure time–distance relationships. It turns out that atomic defects in the surface strongly affect the travelling time with very high sensitivity to their precise location.

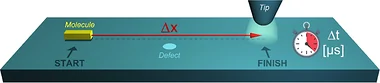

## 1 Introduction

Studying and resolving the motion of objects in time is key to understanding processes in nature, both at the macroscopic scale [1] and the level of atoms and molecules [2]. Understanding molecular motion on solid surfaces is crucial in chemistry, as it provides valuable insights into reaction mechanisms and dynamics [3, 4]. Molecular velocities are important in this regard as they determine the available kinetic energy and thus yield of reaction upon collision. Similar to the Maxwell–Boltzmann distribution, velocities follow a wide range, which are very difficult to determine individually. Such measurements require monitoring of a single molecule along a well‐defined path. Scanning tunneling microscopy (STM) is a suitable method as it can image individual molecules and also allows their manipulation. However, STM typically images distant snapshots, because surface motion is too fast to be captured. This has been improved by fast imaging [5, 6], but the real speed of molecules on surfaces has never been measured.

Typically, molecular motion on surfaces is thermally driven. Molecules move in a random walk, following the Arrhenius equation [7]. Translation occurs in two dimensions [4], but is sometimes confined to one dimension, for example along the rows of a corrugated surface [7, 8, 9] or along the straight standing waves of surface electrons within a nanostructure [10]. One‐dimensional motion was even observed on flat fcc(111) surfaces [11, 12], where molecules follow symmetry directions of the surface lattice.

Even if motion is confined to one dimension, it still occurs in two directions (back‐and‐forth) [13], as the potential energy surface (PES) is periodic. However, for molecular velocity measurements, uni‐directional movement (i.e., only forward and not backward) is needed. This can be achieved by a motor unit [14] or a local gradient that distorts the PES in favor of one direction over the other. In STM, such a gradient can be applied via the local electric field between tip and molecule [15]. During translation, molecules typically hop from one surface lattice site to another [16]. An exception is dibromo‐terfluorene (DBTF) that can travel large distances precisely along a straight row of surface atoms on Ag(111) [12], representing a very suitable system to investigate molecular motion. However, the millisecond time resolution was insufficient to capture details of the molecular translation. Here we use a modified electronics setup (Figure S1) with a thousand‐fold faster time resolution of 1.8 µs, which can resolve the molecular motion.

## 2 Results and Discussion

### 2.1 Time–Distance Relationship of Single‐Molecule Motion

High‐mobility DBTF molecules (Figure 1a) were studied on the Ag(111) surface. They were obtained by orienting DBTF along the close‐packed $\left[1\bar{1}0\right]$ surface direction, following its removal from a molecular island (Figure 1b left). We note that we otherwise find no DBTF in the high mobility configuration, even following deposition on a surface held at 40–60 K (Figure S2), implying a low barrier for conversion to the more stable “immobile” configuration. The resulting high‐mobility species appears as a stripe across the STM image (Figure 1b right), due to the attraction between tip and molecule [12]. We determine the relationship between travelling time and distance of the molecule by setting it into its mobile orientation (“initial position” in Figure 1c,d) and then placing the STM tip at a chosen distance (“final position”) along the same atomic row (dashed line), which represents the pathway length Δ*x* (for details of the procedure see the Supporting Information). By switching the voltage from −1 to +1 V (at time zero; Figure 1e) the molecule is attracted toward the tip. The molecular arrival underneath the tip is detected via a sudden current increase (Figure 1e), due to the reduced tunneling gap. For example, the molecule in Figure 1e (top) takes Δ*t* = 31 µs to move from the initial to the final position.

**FIGURE 1:**
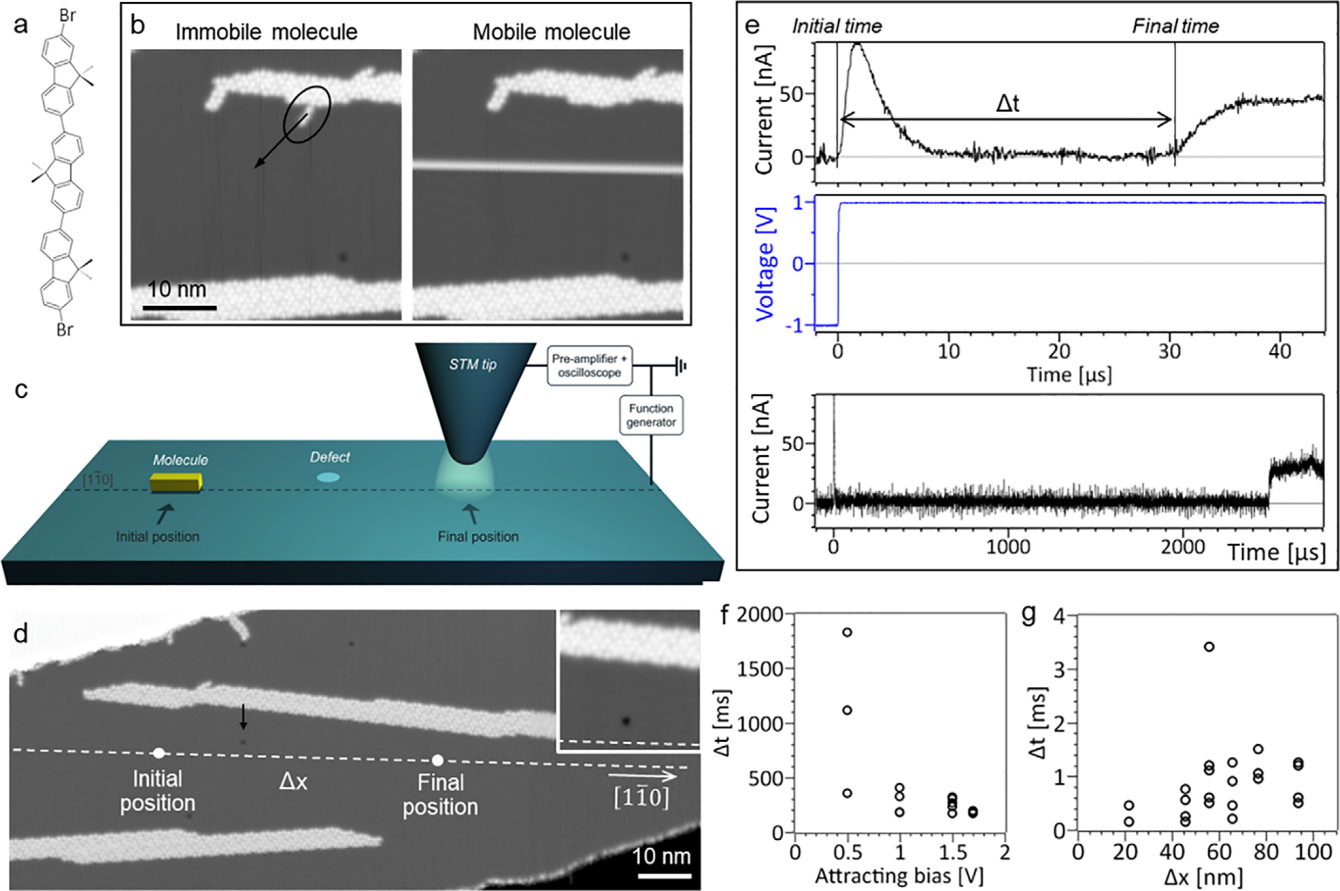
Single‐molecule speed measurement. (a) Chemical structure of DBTF. (b) STM images (0.5 V, 50 pA) of the same surface area before (left) and after (right) bringing a DBTF molecule into its mobile configuration through STM manipulation (black arrow). (c) Scheme for velocity measurements (see Methods in Supplementary Information) with the corresponding STM image (0.5 V, 50 pA) in (d). Distances Δ*x* between initial and final positions are measured along the indicated path (dashed line). Inset: Zoom‐in of the surface defect in the STM image (indicated by a black arrow). (e) Current traces recorded during velocity measurements. Initial and final times are indicated. After an inductive spike between 0 and 10 µs, the current starts to rise at Δ*t* = 31 µs, indicating arrival of the molecule under the tip after travelling 38 nm. The bottom trace shows a longer travel time (∼2.5 ms) for a distance of 104 nm. (f) Δ*t* measured for the same molecule at fixed distance (Δ*x* = 93 nm), but different attracting voltages. (g) Δ*t* measured for the same molecule at varying distances Δ*x* along the path in (d).

The molecular motion is electrostatically driven, whereby the molecular dipole reacts in response to the (inhomogeneous) electric field of the STM junction [12], which we confirmed through changing the electric field strength (Figure 1f). The very same molecule is repeatedly moved along the same track (Δ*x* = 93 nm) at a bias voltage that is increased in each repetition (at constant tip height). Hence, the attracting electric field increases in strength, causing stronger acceleration of the molecule. Accordingly, we find a decrease in the measured interval Δ*t* (Figure 1f), confirming the electrostatic nature of the interaction. Note that, already from an attracting bias of 1 V onward, the spread in the return times is significantly reduced, likely because the molecular motion is then dominated by electrostatic attraction to the tip rather than by inhomogeneities in the potential energy landscape as discussed below.

To determine the time–distance relationship, we measured Δ*t* for increasing Δ*x* values with one DBTF molecule along the very same atomic row (to exclude chemical/structural effects that might occur when comparing molecules in different areas) (Figure 1d). We find that Δ*x* and Δ*t* are related (Figure 1g) since larger distances result in longer travelling times, which is obviously associated to the speed of the molecule. However, the relationship in Figure 1g is not simply linear, as one might expect, which is caused by the precise composition of the pathway as discussed in the following.

### 2.2 Influence of Surface Defects

We observe in Figure 1g that, at each Δ*x*, the distribution of return times Δ*t* is large. Moreover, it appears surprising that the interval Δ*t* can greatly vary for different measurements (e.g., Figure 1e top/bottom), although identical molecules were pulled across flat terraces. In fact, in a systematic comparison, a spread over four orders of magnitude in Δ*t* is found (Figure 2a). This variation can be understood if the pathway of motion is inspected in detail. For instance, in the STM image of Figure 1d, a surface defect, visible as a small black dot (black arrow), is located near the molecular pathway (inset). In fact, distinguishing the data into cases with and without such a defect, an interesting separation occurs: molecules without such a defect travel rather fast (red, solid circles in Figure 2a), while molecules that have defects near their track (within 3 nm of their pathway) move at lower speed (black, open, and solid circles). Note that the data in Figure 2a are presented in a logarithmic plot, spanning several orders of magnitude, to cover the large spread of Δ*t* data points.

**FIGURE 2:**
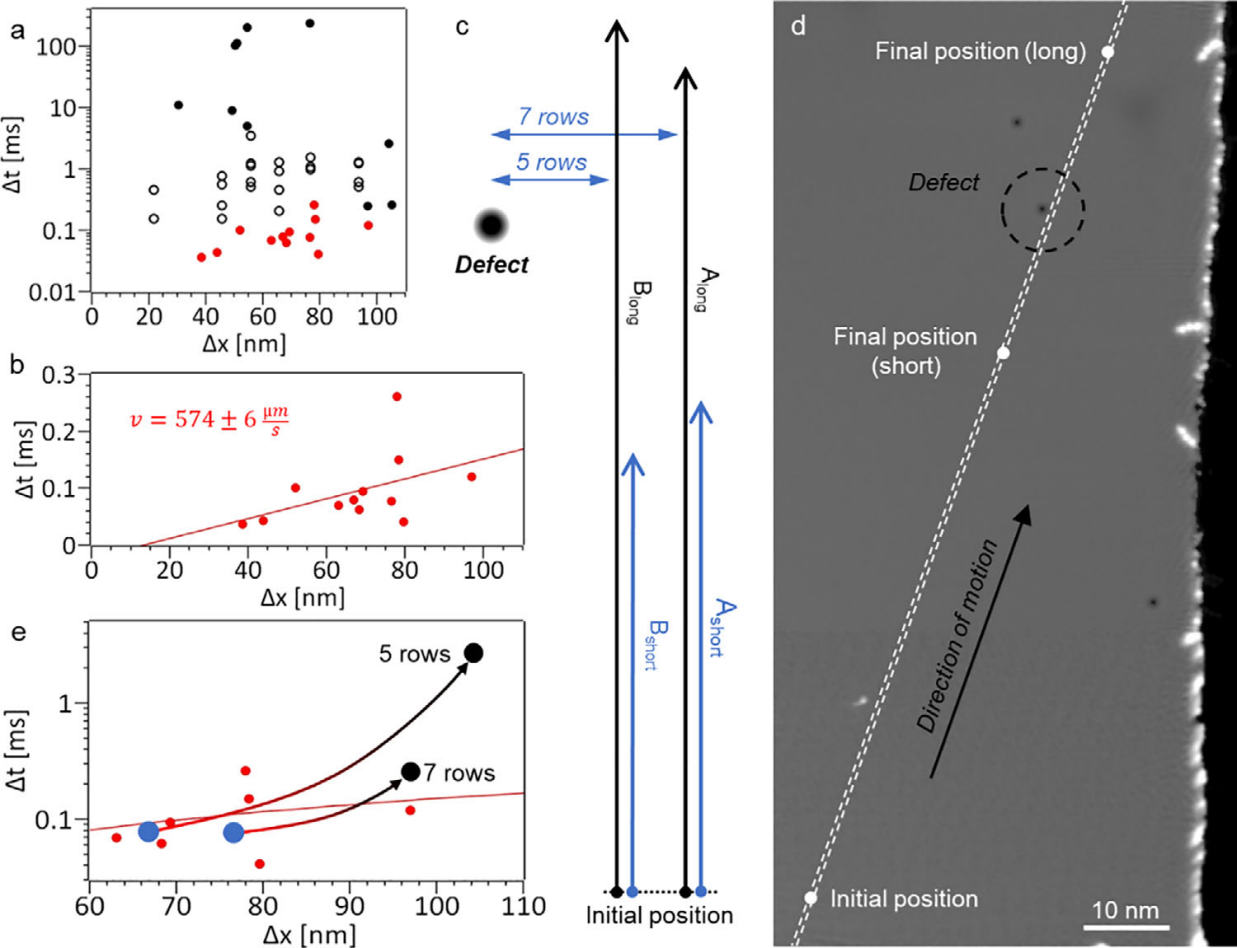
Surface defects change the molecular velocity. (a) Travelling times Δ*t* measured for various travelling distances Δ*x* for molecules on different paths. Open/solid black circles refer to pathways with a surface defect nearby (i.e. closer than 3 nm). The open black circles are the data points from Figure 1g. Red, solid circles correspond to pathways without a defect. (b) Same red data points as in (a). A linear fit is used to determine the average velocity. Note that the (0 nm/0 ms) origin was deliberately not fixed in the fitting to evaluate purely the proportionality of the data. However, the fit (*y*‐axis intercept at −0.02 ± 0.08 ms) yields a standard error that is sufficient to include the origin, which confirms the assumption that the molecule departs from the start of the track (see Methods in Supplementary Information). (c and d) Scheme and STM image of the experiment with two parallel pathways (dashed lines) at distances of 7 or 5 close‐packed silver atom rows (path A or B) from the defect (dashed circle) (see Figure S3). The initial position and two possible final positions are indicated—depending on whether the path includes the defect (long) or not (short). (e) Zoom‐in of plot (b), including results from the experiment described in (c and d) as bold symbols. Blue bold circles are measured on the paths *A*_short_ and *B*_short_ while black bold open circles are measured on *A*_long_ and *B*_long_. Arrows indicate the change in Δ*t*, due to the presence of the defect along the path.

However, Δ*t* values and their spread are strongly reduced if only data points that correspond to molecules travelling on a “defect‐free” pathway (Figure 2b) are considered. A pathway is defect‐free when no defects lie within 3 nm to either side of it (determined from inspection of high‐resolution STM images, similar to the inset of Figure 1d). For these molecules, a roughly linear relation in the time–distance trace is found, indicating that the real velocity of a single molecule is measured here. From a linear fit, an average speed of ∼570 µm/s is determined (Figure 2b). To put this into perspective: this is much faster than the motor protein kinesin that moves with ∼0.8 µm/s along microtubules [17], but also much slower than molecular fragments that desorb at about 3 × 10^9^ µm/s from a surface after CH_3_Br dissociation [18]. However, in contrast to these fragments, which are detected in the gas phase and have velocities comparable to those of gas‐phase photolysis (∼10^9^ µm/s) [18], the molecules in our study remain always adsorbed on a surface. This wide range illustrates that a molecule's speed is a measure of its interaction with the environment and that this interaction is responsible for the differing velocities—as is the case for our findings with surface defects.

The travelling times of molecules with a defect near their pathway not only differ from defect‐free cases but also show a large spread (Figure 2a). We attribute this to variation in the defect's distance from the molecular pathway or even to the presence of multiple defects in some cases. Note that this variation is not caused by the molecule itself, as evidenced by the small spread in data for defect‐free pathways (Figure 2b). The presence of a defect along the molecular pathway affects the travelling time likely in the following manner: we assume that the molecule travels with the defect‐free velocity of ∼570 µm/s in the regions before and after the defect, while the defect itself causes a delay time that adds up as an “offset” to the measured travelling time. Note that this delay time is stochastic in nature (if other effects like e.g., defect distance from the pathway are not considered, see below), causing a substantial spread of the data points. In summary, a roughly linear relationship Δ*t*/Δ*x* can be only observed in defect‐free cases, while data points for pathways that include a defect are shifted upwards to much higher Δ*t* values (red and black circles, respectively, in Figure 2a).

Importantly, the delay time can be tuned in our experiments. We show this in an experiment where the very same molecule is studied and the influence of a defect is modified by using two parallel pathways (A and B) with different distances from a fixed defect (Figures 2c and S3). Additionally, the travelling distance along these pathways is also varied, either with or without a defect next to it: pathway “short” (or “long”) if the “final position” is chosen before (or behind) the defect (arrows in Figure 2c). Figure 2d shows an overview of the two pathways and the one initial and two final positions. This experiment shows that for both cases (A and B) the molecule becomes slower when the defect comes into play, that is, when the pathway is changed from “short” to “long” (arrows in Figure 2e). Hence, the molecule did not only travel a longer distance but was also slowed down by the defect. For pathway A, with the defect at 7 atomic rows distance, the effect is less pronounced, even though Δ*t* still increases by a factor >3 beyond the slope of the defect‐free data. For pathway B, with the defect at 5 atomic rows distance, the effect is much clearer as the travelling time increases by about one order of magnitude. This result reveals the high sensitivity to the precise defect location with respect to the molecular path.

### 2.3 Mechanism of the Molecular Motion

To understand how a surface defect can affect the travelling time of a molecule, one must first know the defect's chemical identity. These defects, visible as dark spots (Figure 1d), are already present on Ag(111) before molecular deposition (Figure S4) and show a bias‐dependent appearance—either as a sombrero‐hat shape at −2 V or a depression at +2 V (Figure 3a). Moreover, they cannot be dislocated by lateral manipulation, even at very small tip heights (Figure S5), therefore we suggest that they are embedded in the first silver layer. Such defects have been observed before and were suggested to be either S or C atoms [19]. Thus, we have performed density functional theory (DFT) calculations for sulfur and carbon embedded in Ag(111), as well as for oxygen—a common contaminant. We find the resulting simulated STM images for sulfur show a very good agreement with the experiments (Figure 3a,b), while carbon and oxygen (Figure S6) do not. Hence, we conclude that the defects are single S atoms embedded in the first Ag(111) layer.

**FIGURE 3:**
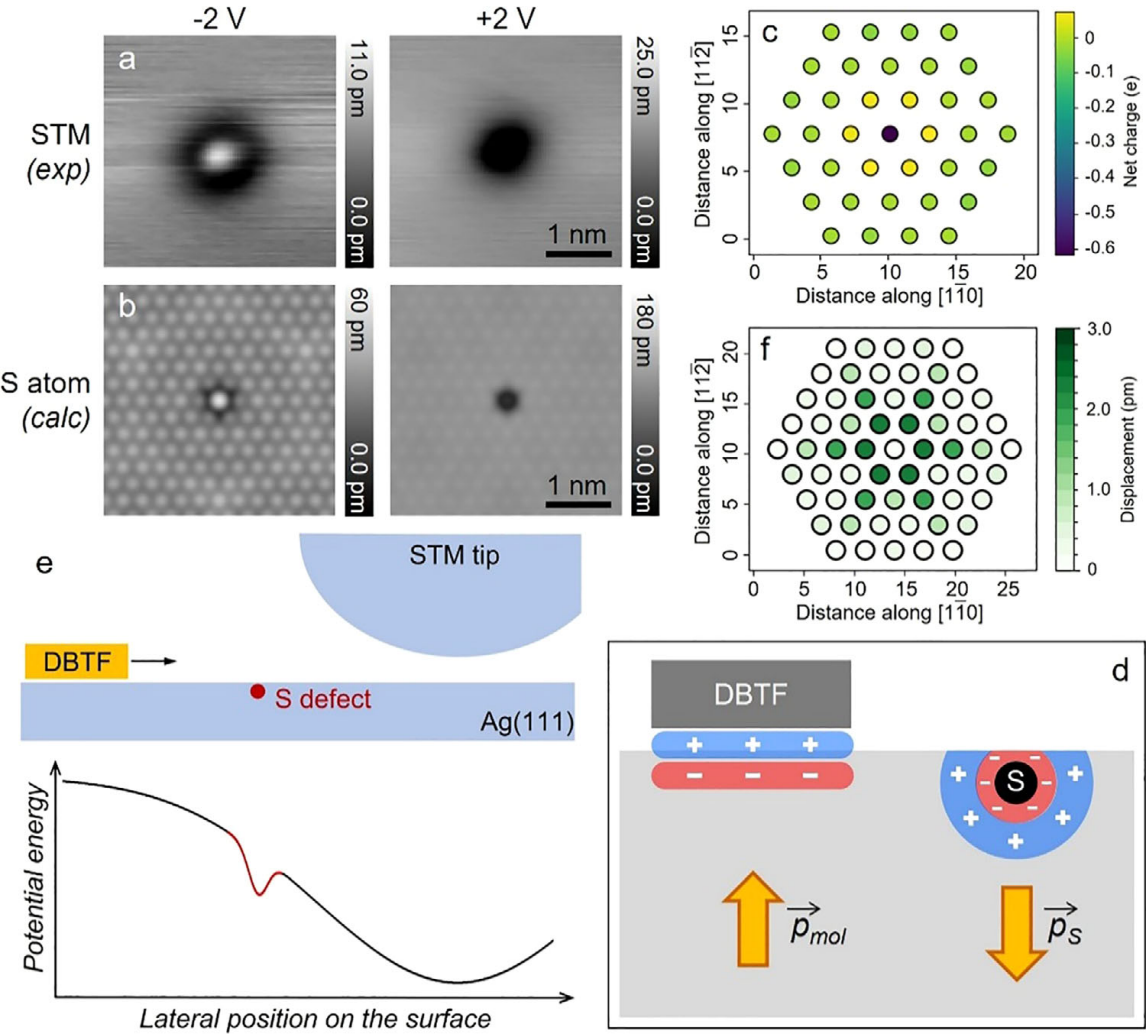
Sulfur atom embedded in top surface layer. (a) Experimental STM images of a defect recorded at −2.0 V and +2.0 V. (b) Simulated STM images of an S atom, substituting an Ag atom of the top layer of a 9 × 9 × 5 slab. (c) Calculated charge distribution of an S atom, substituting an Ag atom of the top layer of a 9 × 9 × 5 slab. (d) Sketch of the electric dipole moments ${\vec{p}}_{mol}$ and ${\vec{p}}_{S}$ that are present at the position of the DBTF molecule and the S defect, respectively. (e) Illustration of how the S atom affects the motion of the molecules via a modification of the potential energy landscape at the position of the S defect. For simplicity, the relatively small surface corrugation, which leads to a washboard potential [23], is not included here (see Figure S7 for a more detailed sketch). (f) Calculated in‐plane displacements of the atoms in the first surface layer, smaller values are obtained for the layers underneath and for out‐of‐plane displacements (see Figure S8).

The key question is now how a single S atom can affect the molecular motion nearby. We consider two mechanisms: First, the S atom could locally distort the Ag(111) lattice, thereby modifying the PES and influencing the molecular motion. Such an effect has been described theoretically for adatom diffusion [20]. Experimentally, it is known that the arrangement of surface atoms affects the adsorption energetics of molecules, for example, adsorption on fcc and hcp domains of Au(111) [21]. We therefore calculated the extent to which a sulfur atom distorts the silver lattice and found a maximum deviation of 2.3 pm in the lattice constant (Figure 3f). However, this effect is not only very small in magnitude, but also very local—only those silver atoms within 7.5 Å (i.e., three lattice sites) from the defect show a deviation in their lattice constant. This contrasts with our experiments, where the effect is also present if the molecular pathway is 2 nm away from the sulfur. Therefore, lattice distortions are unlikely to be responsible for the increased travelling time.

Second, the sulfur atom locally modifies the charge distribution in the surface. DFT modelling of the charge distribution shows a local accumulation of about −0.6e at the S atom (Figure 3c). This accumulation of negative charge, due to the electronegativity of sulfur, results in a vertical dipole moment ${\vec{p}}_{s}$ that points downwards (sketched in Figure 3d). Hence, it is opposed to the electric dipole moment ${\vec{p}}_{mol}$ at the interface between the DBTF molecule and the silver surface [12, 22]. Since the two dipole moments are oriented in opposite directions (Figure 3d), the molecule is attracted by the sulfur atom. Consequently, the molecule that is pulled across the surface by the electric field of the STM tip experiences a local dip in the PES (Figure 3e). This localized PES deformation reflects the local attraction experienced by the molecule, resulting in slower motion. It also explains pathways A and B in Figure 2d: the closer the molecular trajectory is to the S atom, the deeper the potential well and, consequently, the longer the time to escape from local trapping.

### 2.4 Periodic Motion in an Oscillating Field

The observed motion is very reliable—it can be repeated many times with a molecule traveling back and forth between the STM tip and some remote location along the same pathway. This is demonstrated by applying a bias voltage that oscillates at a frequency *f* between a positive and a negative value (blue curve in Figure 4b). This results in alternating attractive and repulsive electrostatic forces that send the molecule away from the tip and pull it back, respectively (Figure 4a). The tunneling current at constant tip height illustrates this by oscillating with *f* between a large positive and a small negative value (Figure 4b). In the former case the molecule is located in the STM junction, causing a high current, while in the latter the molecule is remote from the tip, resulting in an “empty” junction and a low current. This can be reproducibly done over many (even thousands, Figure S9) oscillations.

**FIGURE 4:**
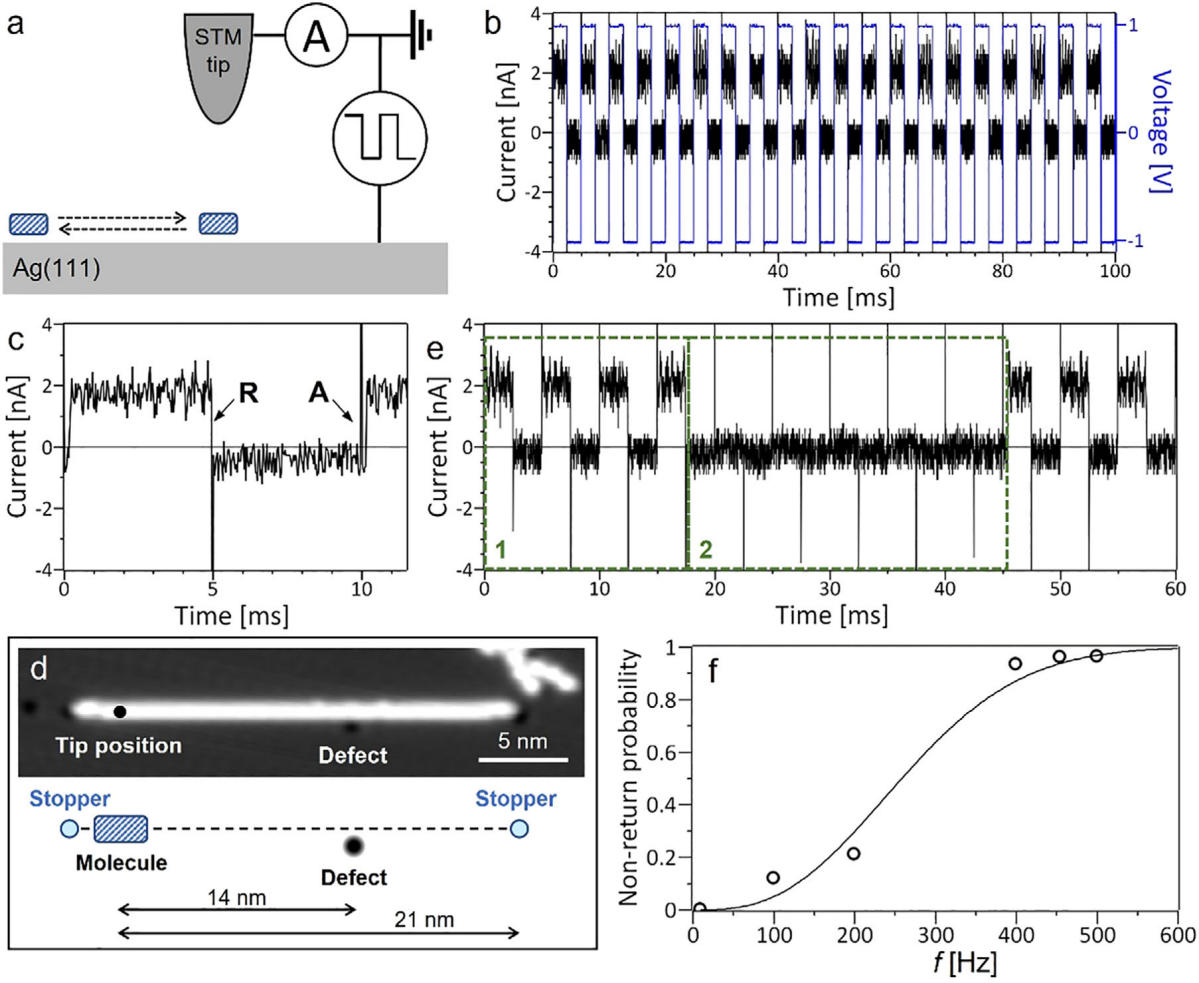
Oscillating molecular motion. (a) Setup for the alternating bias voltage experiment: a square wave voltage is applied to the sample, and the molecule moves in/out of the tunneling junction, depending on the polarity. (b) Applied bias signal (blue; ±1 V, 200 Hz) and tunneling current response (black). (c) Single‐cycle current trace with the molecule under the tip at +2 nA (+1 V) and outside the junction at −0.1 nA (−1 V). Each bias switch causes a short inductive current spike. R and A indicate the start of repulsion and attraction, respectively. (d) STM image of an alternating bias experiment with the corresponding scheme below. Molecular motion is constrained between two surface defects (“Stopper”), another defect lies along the pathway. All three defects are identical (S atoms), but those at the termini cannot be surpassed by the molecule. The defect at the side is very close (4 atomic rows away) and so affects the molecule even more strongly than in Figures 1d or 2c, as evidenced by the altered stripe appearance near the defect (see also Figure S3). (e) Current trace under alternating voltage (±1 V, 200 Hz) with the tip position from D. Two regimes appear (dashed boxes): (1) the molecule responds to the bias polarity as in B–C; (2) the molecule remains away (low currents). (f) Non‐return probability (line is a visual guide). It is 1 if the molecule is trapped behind the defect and 0 if it returns under the tip at every cycle.

The repulsion of the molecule is visible as an abrupt decrease in the tunneling current, indicated as R (for “repulsion”) in Figure 4c. The molecule is first located underneath the tip and then quickly moves out of the junction when the bias changes from positive to negative. Accordingly, the tunneling current signal immediately follows the voltage signal at rather large STM tip heights. At small tip heights, a delayed departure of the molecule occurs, due to van der Waals trapping [23] in the junction (Figure S10). After the molecular departure the current remains close to zero, reflecting the “empty” STM junction. Then, in the second half‐cycle, the polarity is reversed and the molecule returns into the junction (A for “attraction” in Figure 4c). The travelling time of the molecule to reach the tip is manifested as a delay in the tunneling current jump, which can be measured (Δ*t* = 0.25 ms in Figure 4c). Note that these measurements do not simply average over many events, but resolve every single event.

To get more insight into this process, a specific pathway was used, which is terminated at both ends by sulfur defects (“Stopper”) and has an additional defect next to it at a very close distance of 4 atomic rows (Figure 4d). The STM tip is then placed at the left end of the pathway (“Tip position”) and square wave voltages with various frequencies *f* are applied (tip at constant height). With the defect so close to the pathway two regimes can be distinguished (Figure 4e): (1) the molecule follows the bias oscillation, moving in and out of the STM junction and a return time can be measured for each cycle if the length of the free pathway before the defect is long enough (see Figure S11 for details). (2) The molecule is always remote, resulting in a current close to zero. The latter corresponds to a situation in which the molecule has surpassed the defect and remains trapped “behind” it (i.e., at the right end of the pathway in Figure 4d) and does not return to the tip in every cycle. The return happens only occasionally (e.g., at 45 ms in Figure 4e) when regime (1) is restored.

This interpretation is confirmed by tuning the frequency *f*, which modulates the relative abundances of regimes (1) and (2). The process is quantified as “non‐return probability”, which is the number of cycles in which the molecule does not return to the tip, divided by the total number of cycles. This value increases for *f* between about 200 and 400 Hz (Figure 4f), that is a period between 5 and 2.5 ms. Since the pathway is well known (Figure 4d), the travelling distance of the molecule and the time intervals can be connected (longer distances display a similar behavior and were used for statistics; Figure S11).

From this transition regime (200–400 Hz), we estimate that the molecule needs between 1.25 and 2.5 ms (i.e., half period, during which the tip is attractive) to travel from behind the defect to the tip. If *f* < 200 Hz, the molecule has more time in the attractive regime and can return more often. If *f* > 400 Hz, the molecule does not have enough time to return under the tip. The travelling time along the free path is negligible, since the molecule moves at the “free” velocity (574 µm/s was measured in Figure 2b) for 14–21 nm (minimum–maximum distance from behind the defect to the tip position, see Figure 4d). Therefore, the measured travelling time (1.25–2.5 ms) is dominated by the delay at the defect. The larger delay observed here, compared to the previous case, is consistent with the defect being located closer to the molecular path (4 atomic rows).

These measurements (with a fixed STM tip position, no tip repositioning, or a multi‐probe instrument required) contain many translations of the oscillating molecule, thus providing better statistics, and confirm the results obtained with a single bias switch. For better insight, we also measured the return times of the molecule in regime (1) with an extended pathway (Figure S11). This was done under the same conditions as in Figure 4d, but without stoppers and for different distances. Histograms of the return times reveal a Poisson distribution, suggesting that the molecular departure from the potential well is a stochastic process.

## 3 Conclusion

Our study gives fundamental insight into molecular motion on a surface. It shows how local perturbations can slow down or even trap molecules, which is relevant for on‐surface growth and polymerization and in heterogeneous catalysis where atomic defects are important [24]. Furthermore, the capability to steer molecular motion via controlled implementation of atomic defects or alternating electric fields might open avenues for the nanoscale transport of matter.

## Author Contributions

**Donato Civita**: conceptualization, experiments, formal analysis, and writing. **Matthew J. Timm**: calculations, editing, and funding acquisition. **Julia Lanz**: experiments. **Christophe Nacci**: experiments and editing. **Stefan Hecht**: providing molecules and editing. **Leonhard Grill**: conceptualization, formal analysis, writing of the original draft, editing, project administration, supervision and funding acquisition.

## Conflicts of Interest

The authors declare no conflicts of interest.

## Supporting Information

The authors have cited additional references [S1–S22] within the Supporting Information.

## Supporting information




**Supporting File**: anie74643‐sup‐0001‐SuppMat.pdf.

## Data Availability

The data that support the findings of this study are available from the corresponding author upon reasonable request.
