# Supplementary material for: Speed Control of Single Molecules on a Surface
Source: Angew Chem Int Ed Engl. 2026 Sep 8;65(44):e5538086. doi: 10.1002/anie.5538086 (PMC13703937; doi:10.1002/anie.5538086)
Supplement: Supplementary file 1 [file ANIE-65-e5538086-s001.pdf]

Supplementary Information

Speed Control of Single Molecules on a Surface

Donato Civita[1], Matthew J. Timm[1], Julia Lanz[1],
Christophe Nacci[1], Stefan Hecht[2], and Leonhard Grill[1*]

[1]*Department of Physical Chemistry, University of Graz, Austria*
[2]*Department of Chemistry and IRIS Adlershof, Humboldt-Universität zu Berlin, Germany*
* Corresponding author: leonhard.grill@uni-graz.at

Contents

Methods **S2**
S1. Measurement setup **S4**
S2. Deposition of molecules at cryogenic temperature **S5**
S3. Molecular stripe at different distances from the defect **S6**
S4. Ag(111) surface defects **S8**
S5. Manipulation of surface defects **S9**
S6. Simulated STM images of surface defects **S9**
S7. Washboard potential **S10**
S8. Calculated atomic displacements from a sulfur atom defect **S11**
S9. Examples of current traces **S13**
S10. Delayed departure **S15**
S11. Alternating bias voltage at different distances from the defect **S16**
References **S18**

Methods

Synthesis

Dibromo-terfluorene (DBTF) molecules were synthesized as reported previously.[S1]

Sample preparation

The Ag(111) surface was cleaned by repeated cycles of Ar^+ ion sputtering at 1.25 keV followed by annealing at 720 K. DBTF molecules were deposited by sublimation from a Knudsen cell (at 400 K) onto a Ag(111) sample that was kept at room temperature (deposition time: 30 s). This preparation resulted in close-packed and elongated molecular islands, with large areas of the Ag(111) surface remaining empty.

STM experiments

All STM experiments were done under ultrahigh vacuum with a base pressure of 10^{-10} mbar and at a sample temperature of 7 K. The STM tip was made from an electrochemically etched platinum-iridium wire and then shaped by soft indentation into the Ag(111) surface at bias pulses of few volts. Imaging was done in constant-current mode by applying the bias voltage to the sample (tip grounded). STM manipulation of molecules was done in the constant-current mode with $U = 0.1$ V and $I = 10$ nA.

Velocity measurement experiments

Velocity measurement experiments (sketched in Fig. 1c) were carried out with a single molecule starting at the 'initial position' and aligned with the $[1\bar{1}0]$ symmetry direction (dashed line), *i.e.* in its mobile orientation.

In the case of measurements with a single bias switch we proceeded as follows:

1. STM imaging of the molecular stripe with 0.5 V and 50 pA.
2. Feedback is deactivated, therefore the tip position remains fixed, and the bias is set to zero.
3. The STM tip is first retracted vertically by hundreds of nanometers and then laterally positioned at the chosen 'final position' above the path of the molecule. Note that, to ensure that the DBTF is not displaced from its 'initial position' during tip repositioning, the tip takes a large path around the molecular path to reach the 'final position'. When the tip is positioned above the molecular pathway, the feedback is reactivated with -1.0 V and 50 pA to re-approach the surface.
4. Feedback is deactivated again. The molecule is in this moment still at its initial position or in close proximity to it. If it would move by itself over longer distances during steps 1-4, it would likely be found adsorbed at the next step edge, which represents a stable adsorption site. This never happened. Moreover, the real travelling distances (during step 5) would vary a lot, so would the measured travelling times, which is not the case in Fig. 2b.
5. Bias supply switched to the function generator, which switches the bias to +1 V. The tunneling current is measured with an oscilloscope.
6. Bias supply switched back to the STM electronics, which applies again -1 V.
7. Feedback reactivated with 0.5 V and 50 pA and STM imaging of the molecular stripe at the new location is performed.

For the measurements with alternating bias voltage:

1. The STM tip is positioned above the molecular stripe.
2. Feedback is deactivated, *i.e.* the tip position remains fixed, and a bias of +1 V is applied.
3. Bias supply is switched to the function generator, which applies the alternating bias voltage at the desired frequency and bias values. The tunneling current is measured with an oscilloscope.
4. Bias supply is switched back to the STM electronics, which applies again +1 V.
5. The feedback is reactivated with 0.5 V and 50 pA.

A large-scale STM image of the entire path is also recorded to identify any surface defects along the trajectory. The bias is applied to the sample using a function generator (single bias switch or alternating bias voltage), while the tunneling current is measured from the tip with a preamplifier and an oscilloscope (see Fig. S1 for details of the measurement setup).

Linear fitting

The linear fitting in Fig. 2b is done by least square minimization using the function: $t(x) = t_0 + \frac{1}{v}x$ where v, which is the velocity, and t_0 are the two free parameters. The parameters uncertainty is obtained from the covariance matrix and defined as (Upper Bound - Lower Bound) / 2, where Upper Bound and Lower Bound are the upper and lower limits of the confidence interval, respectively. The confidence intervals are determined from the standard error of the parameters. The standard error of the parameters is the square-root of the diagonal of the parameter covariance matrix.

Theory

Density Functional Theory (DFT) calculations were performed using the Vienna Ab initio Simulation Package (VASP)[S2-S3] version 6.4.3 installed on the Graz Scientific Cluster (HPC GSC1). These calculations used the projector augmented wave (PAW) method[S4-S5] and the generalized gradient approximation (GGA) with the Predew-Burke-Ernzerhof (PBE) functional.[S6] Grimme's semi-empirical dispersion correction (DFT-D3) was added to correct for Van der Waals interactions.[S7] The cut-off energy for the plane-wave basis set was 400 eV.

The Ag(111) surface was modelled by two periodically repeated slab models consisting of: a 9 × 9 supercell with 5 atomic layers (405 Ag atoms) and a 12 × 12 supercell with 3 atomic layers (432 Ag atoms), with each having a vacuum region of 20 Å. To model the embedded atoms, a single Ag atom in the top layer of the slab was replaced by an S, C or O atom. All atoms were allowed to relax until the force on each atom was less than 0.01 eV·Å^{-1}, except for the lowest two Ag layers, which were frozen at the theoretically optimized Ag lattice constant of 4.0742 Å. A single Γ-point *k*-mesh was used for structural optimizations, STM-simulations, and atomic orbital projected density-of-states (pDOS) calculations with Gaussian smearing of $\sigma = 0.25$ eV. The STM simulations were performed using the Tersoff-Hamann approximation[S8] and were visualized using the Hive Software.[S9-S10] Bader charge analysis was performed using the Bader charge analysis code version 1.05.[S11-S13]

S1. Measurement setup

The schematic representation of the measurement setup is shown in Fig. S1a. A bias voltage is applied to the sample with respect to the grounded STM tip, either via the STM electronics or by a function generator, with a switch alternating between these two inputs. The STM electronics are used to bias the sample during scanning, though the STM electronics cannot provide a bias switch or alternating square-wave bias defined at the microsecond resolution. Therefore, for the velocity measurement experiments we use an external function generator that can provide a bias switch between two values with a very short rise time of 0.3 µs (see centre of Fig. 1e in the main text). This very precise bias switch defines also a precise time for the start of the attraction or repulsion, and therefore the motion of the molecule. Switching between these two bias voltage sources (STM electronics or the external function generator) is done after fixing the lateral and vertical position of the tip.

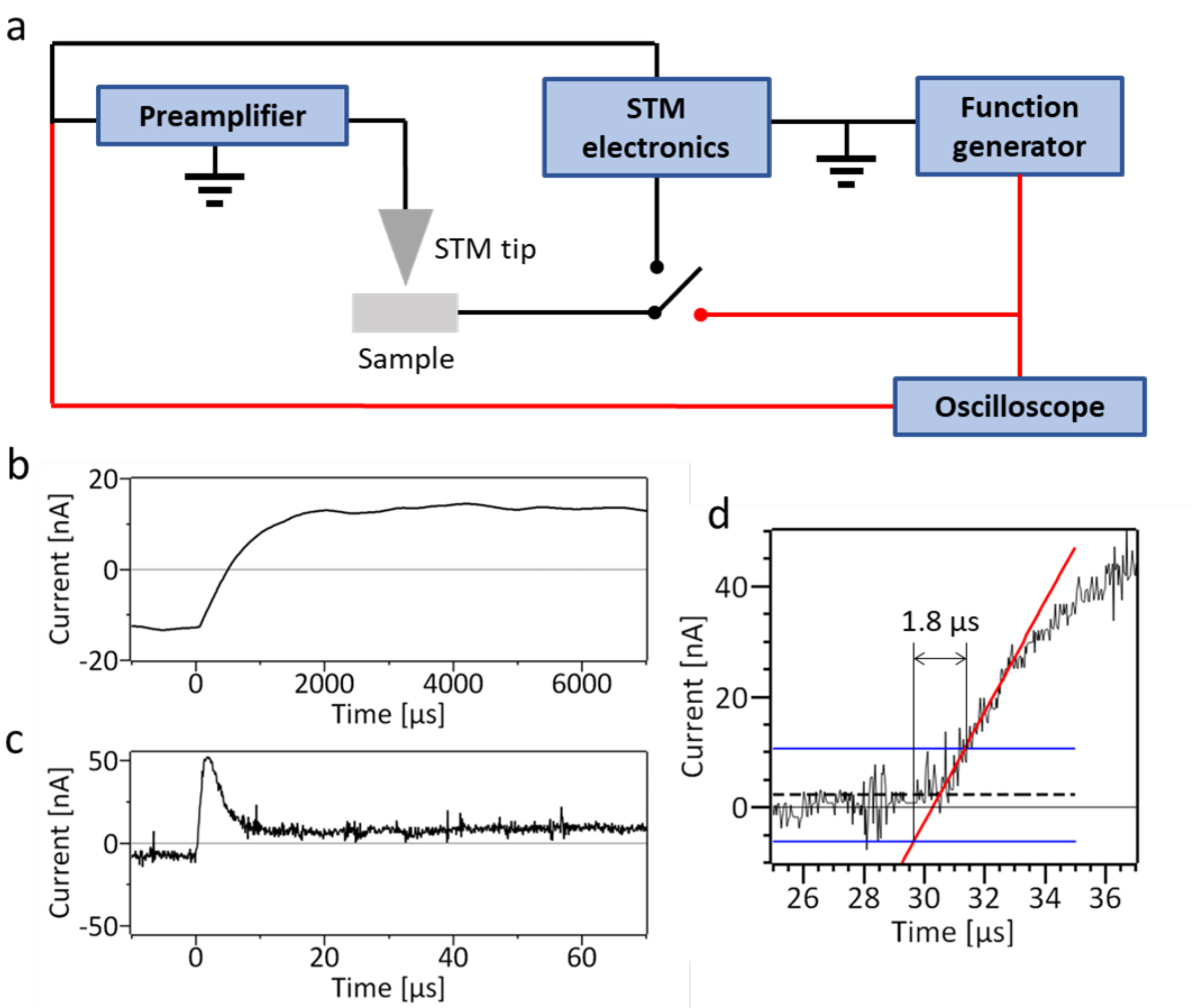

Figure S1. *(A) Schematic representation of the measurement setup. The bias voltage (with respect to the grounded STM tip) is applied to the sample by the STM electronics (black circuit) or by a function generator (red circuit), which can be chosen by a switch. The output of the preamplifier is recorded both by the STM electronics (black circuit) and by an oscilloscope (red circuit). (b-c) Current-vs-time measurement done with (b) the STM electronics or (c) the function generator and oscilloscope showing about three-orders of magnitude difference in rise time for the two set-ups. (d) Current signal before and after the arrival of a molecule under the tip. A dashed black line marks the average current before the molecule arrives, the red line is a linear fit to the rising current. Blue lines indicate our confidence level for detection of current increase, which is three times the standard deviation of the current signal. This gives 1.8 µs (or ± 0.9 µs) as the time resolution of our setup.*

The tunneling current is measured by the current preamplifier (FEMTO variable gain low noise transimpedance amplifier DLPCA-200) and converted into a voltage signal, which is split and then recorded both by the STM electronics and by an oscilloscope (Keysight DSOX3014A Digital Storage Oscilloscope). If the STM electronics are used to both apply a bias switch from -1 V to +1 V and read the current response (corresponding to the black electric connections in Fig. S1a), a current-vs-time trace is obtained like that shown in Fig. S1b. With this setup we are limited in measuring changes in the tunneling current by the rise time of 1000 μs, as visible in Fig. S1b. However, if the function generator is used instead to apply a bias switch from -1 V to +1 V and the oscilloscope is used to read the current response (corresponding to the red electric connections in Fig. S1a), a current-vs-time trace like that shown in Fig. S1c is the result. The sudden spike in the current following the bias switch at 0 μs is due to an inductive effect, caused by the abrupt change in the applied bias voltage and not related to the molecular motion. The sampling rate of the oscilloscope used in this setup is 100 MHz. With the latter setting the rise time is then limited only by the gain of the preamplifier: for example, if the preamplifier is set in low noise mode with a gain of 10^6 V/A, the resulting rise time in the current signal is 1.8 μs (Fig. S1d).

In Fig. S1d (which is an enlarged plot of Fig. 1e) we show that the confidence range for the time the molecule arrives under the tip corresponds to the rise time of the preamplifier. We do this by considering the current value before the molecule arrives beneath the tip, and after. In the first part (black-dashed line) the tunneling current has an average value of 2 nA and standard deviation of 3 nA. At the arrival of the molecule, the increasing tunneling current is fitted with a linear function (red line). The upper and lower bounds for detection of the current increase are indicated by blue lines, given by the average current plus or minus three times the standard deviation, respectively. If we consider the time interval where the red line intersects these upper and lower bounds, we obtain a confidence range of 1.8 μs, which corresponds to the rise time of the preamplifier with a gain of 10^6 V/A. Therefore, with our setup we can measure delay time of a sudden current change larger than 5 μs with a precision of ±0.9 μs. Note that this setup allowed to measure the travelling time for all the cases described in Fig. 1 and 2, since no molecule was found with a traveling time shorter than 30 μs.

S2. Deposition of molecules at cryogenic temperature

We also deposited DBTF molecules onto the Ag(111) sample when it was placed on the manipulator that was cooled with liquid Helium. It was not possible to measure the exact temperature of the sample, while the temperature measured on the manipulator indicated a value of 10 K. The actual temperature of the sample during molecule deposition was some tens K higher, so about 40-60 K. In Fig. S2 we show an overview STM image (taken at liquid Helium temperature) of the sample after this preparation. Single molecules are not observed, instead, they form small, branched clusters of two to ten molecules. This observation indicates that diffusion and molecular reorientation are still possible at the temperature at which the sample was maintained during molecular deposition. If this was not the case and the molecules adsorbed in a 'hit-and-stick' mode, many individual, separated DBTF molecules would be visible. Importantly, we did not find any DBTF molecule in the high mobility configuration after this preparation (*i.e.* no stripes visible), which indicates a low energy barrier for conversion to the more stable 'immobile' configuration after adsorption.

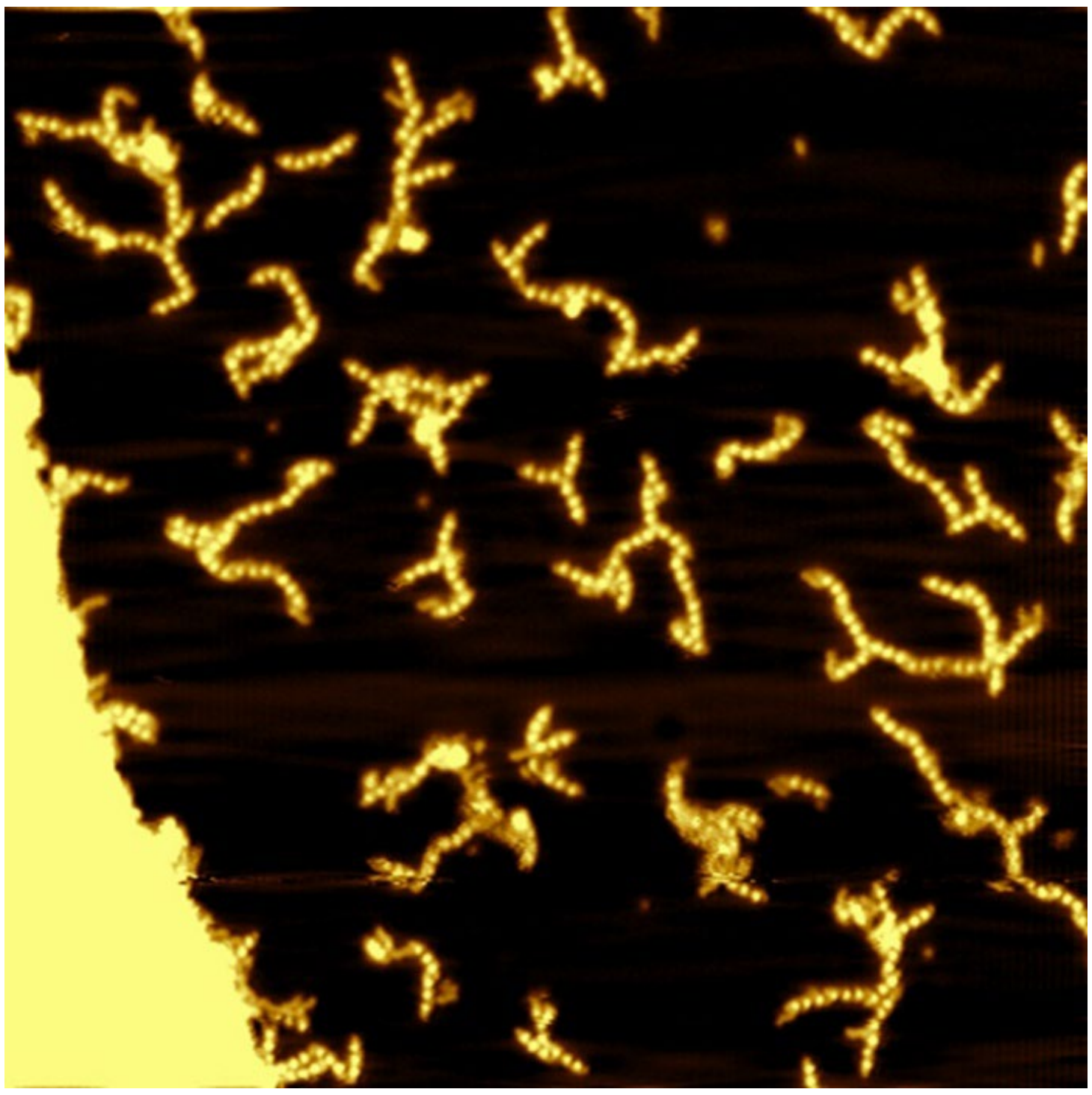

Figure S2. *STM image (0.6 V, 300 pA, 70 nm × 70 nm) of DBTF molecules on Ag(111) surface. The deposition of the molecules was done with the substrate held at temperature lower than liquid nitrogen temperature and higher than liquid helium temperature. On the lower-left corner a step edge of the Ag(111) surface is visible.*

S3. Molecular stripe at different distances from the defect

In Fig. S3 a molecular stripe is presented at different distances from a sulfur defect. The orientation of the dimethyl groups is known for every image from the asymmetric shape of the height profile across the stripe. In all images of Fig. S3, the molecule is pointing with its central dimethyl side group to the left (as indicated in Fig. S3e). This can also be seen in Fig. S3d-e as the molecule stops at the defect.

To define the distance between the molecular stripe and a defect, we take the maximum of the asymmetric stripe's profile as our reference, since it can be clearly distinguished in each STM image. It is indicated in Fig. S3 as a black (vertical) line and is located between two silver rows, one is at 0.10 nm on the left and the other is at 0.15 nm on the right (Fig. S3f). The defect-stripe distance is measured in the (horizontal) $[11\bar{2}]$ direction between the black line and the minimum of the surface defect (white arrow). For example, the distance between stripe and the defect that blocks the path in Fig. S3e is measured as 0.10 ± 0.05 nm, which is the closest atomic row of the substrate on the left side of the stripe. We define this case as the defect being on the first silver row from the molecular stripe (see Fig. S3f).

As the distance between close-packed rows of silver atoms is 0.25 nm (Fig. S3f), the following formula can be used to calculate the distance *x* between stripe and defect: $x = (0.25\ \text{nm}) \cdot (n - 1) + 0.10\ \text{nm}$, where *n* is an integer number starting from 1 (*n* = 1 for the first silver row as in Fig. S3e). This formula is valid if the central dimethyl group of the molecule points to the side of the stripe on which the defect is located (as in Fig. S3, both are on the left side). Otherwise, $x = (0.25\ \text{nm}) \cdot (n - 1) + 0.15\ \text{nm}$ (*i.e.* if the defect would be at the right of the stripe in Fig.

S3f). Thus, the defect in Fig. S3e is located on the first (*i.e.* nearest) close-packed atomic row to the molecular stripe (n = 1), the defect in Fig. S3d on the next nearest (*i.e.* second) row (n = 2), the defect in Fig. S3c on the fourth nearest row (n = 4), the defect in Fig. S3b on the fifth row (n = 5) and the defect in Fig. S3a on the seventh atomic row on the left of the stripe (n = 7). The slight discrepancy between the measured and expected values in Fig. S3b and c is due to the presence of the molecule nearby.

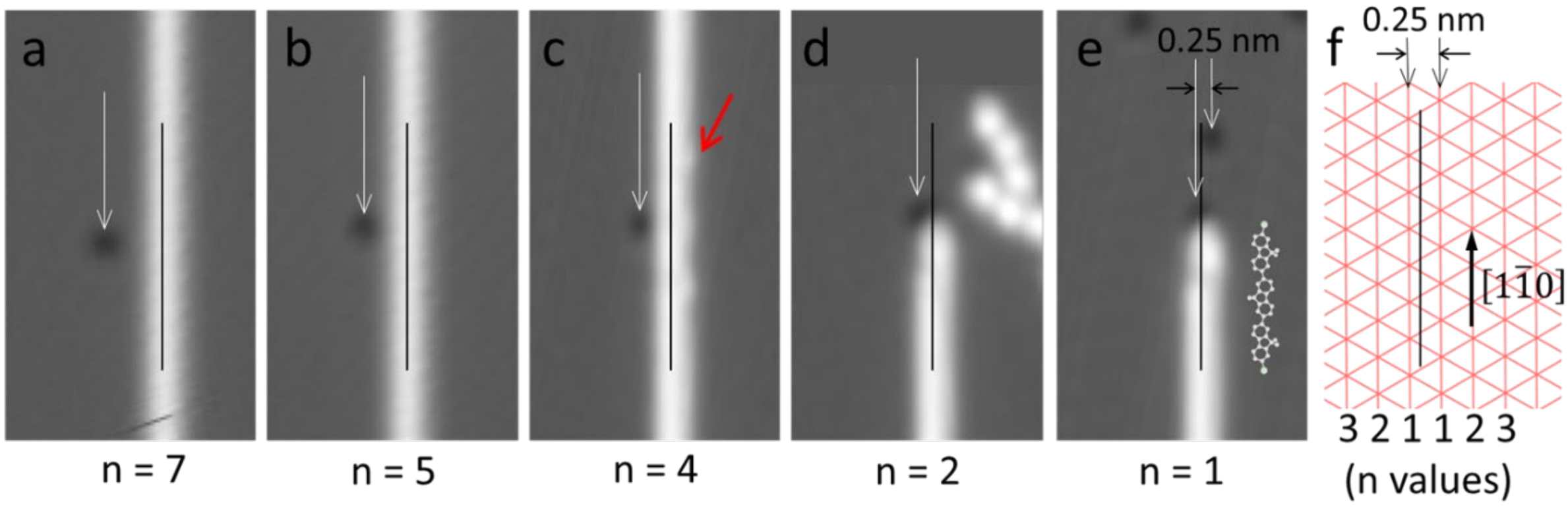

Figure S3. *(a-e) STM images of a molecule in the mobile orientation that moves nearby a surface defect. From (a) to (e) the measured distance (with an error of ±0.05 nm) between the maximum of the stripe profile (black line) and the center of the defect (white arrow) is: 1.62 nm (corresponding to 7 silver rows on the left of the stripe; n = 7), 1.17 nm (n = 5), 0.93 nm (n = 4), 0.39 nm (n = 2), 0.10 nm (n = 1). (a) and (b) were recorded with 0.7 V and 44 pA, while (c-e) were taken with 1.0 V and 400 pA. All images are 7 nm × 12 nm in size. In (c) the red arrow indicates the features on the stripe that appear only at this close distance. Other (immobile) DBTF molecules are visible in (d) on the top right. (e) The distance between the two defects in (e), orthogonally to the* $[1\bar{1}0]$ *direction, is 0.25 nm. The chemical structure of the molecule is superimposed and another defect is indicated by a white arrow. (f) Atomic lattice of the Ag(111) surface, where every cross point is the position of a silver atom. The distance between nearest atomic row of the* $[1\bar{1}0]$ *close-packed direction is 0.25 nm, as indicated. The black line shows the position of the maximum of the stripe as obtained from (e). At the bottom, the n values for the different possible positions of a defect on silver row are given.*

In Fig. S3a-b the profile of the stripe is smooth, including the part where the stripe is closest to the defect. In Fig. S3c the molecule moves on a path which is closer to the defect (0.93 ± 0.05 nm away) and the stripe no longer appears smooth in vicinity of the defect, but exhibits slight protrusions instead (red arrow). At this distance, the interaction between the defect and the molecule weakly traps the molecule as it moves past the defect – resulting in the features visible in the STM image. When the defect is even closer to the molecular path, it completely blocks the motion of the molecule (Fig. S3d-e).

S4. Ag(111) surface defects

The Ag(111) surface is cleaned by Ar^{+} ion sputtering and annealing cycles. The terraces are found to be atomically flat, and the step edges are free from adsorbates. However, small amounts of defects are present that always occur even for a 'clean' Ag(111) surface. The most common defects (indicated with black circles in Fig. S4a) appear as dark depressions, and it is these defects that affect the velocity of DBTF molecules travelling nearby.

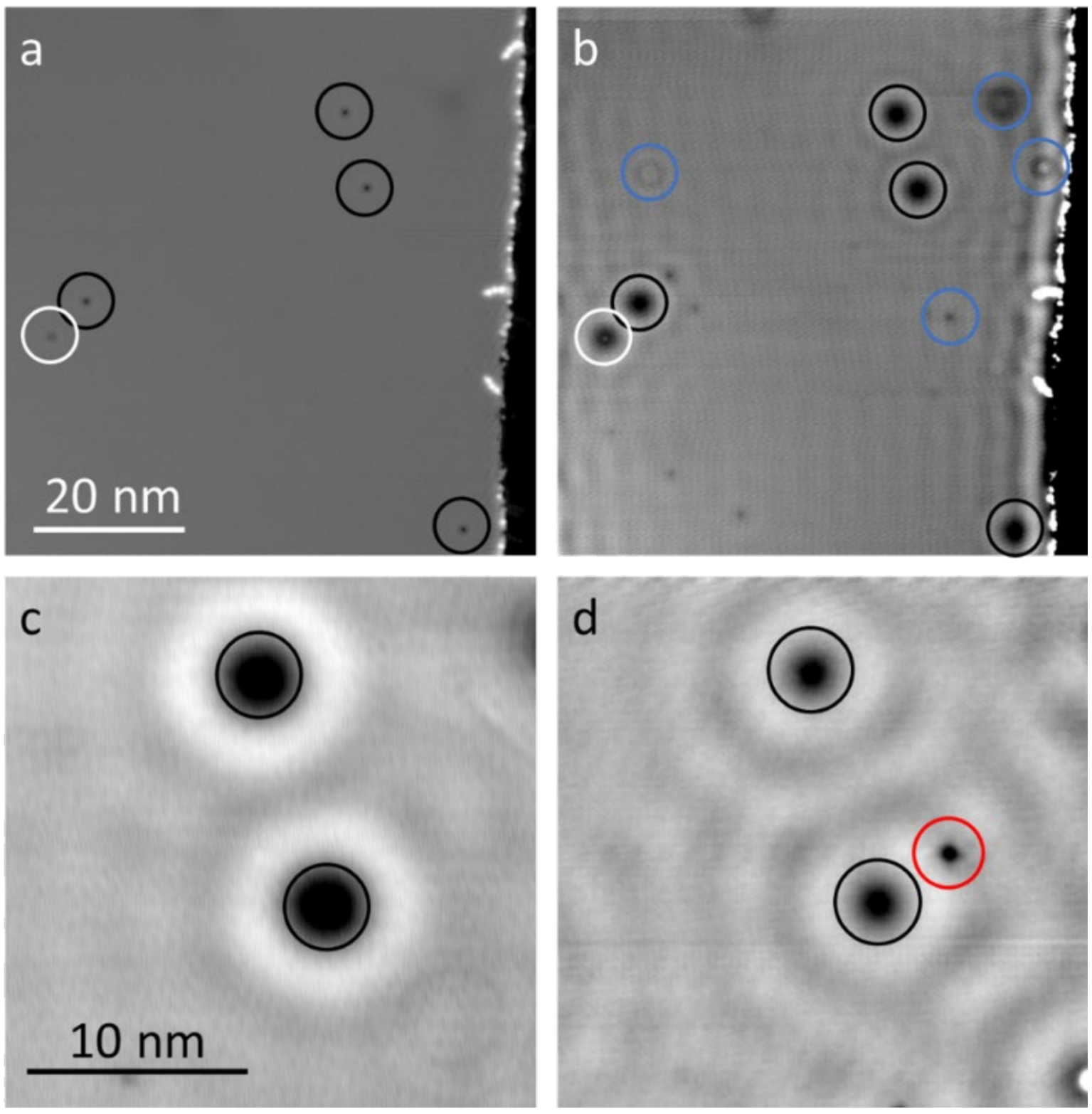

Figure S4. *(a-b) STM images of the same surface area where different dark defects are visible, using different tunneling parameters ((a): 700 mV, 100 pA, (b): -50 mV, 400 pA). Black circles indicate four defects of the same kind, while the white circle indicates a differently appearing dark defect which is probably of another origin. Blue circles in (b) indicate additional defects visible at low bias. (c-d) STM images ((c): -50 mV, 200 pA; (d): 30 mV, 48 pA) of the same area (c) before and (d) after deposition of CO. The previously present dark defects (black circles in (c) and (d)) appear distinctly different from the deposited CO molecule (red circle in (d)).*

We observe that surface electrons are strongly scattered by these defects, creating interference patterns that become visible at low tunneling voltages (Fig. S4), and imaging these defects at very high tunneling resistance (about 330 kΩ) does not induce their displacement (see Fig. S4). These defects are clearly distinguishable by STM from other dark defects (like the one marked by white circle in Fig. S4a) and are also distinguishable from deposited CO molecules (as demonstrated in Fig. S4c-d before/after deposition of CO molecules). Literature suggests that common Ag(111) surface defects are atomic substitutions on the top layer of the crystal due to bulk impurities that segregate to the surface and have been assigned to be presumably either S or C atoms.[S14-S17]

In addition to the aforementioned dark defects, several other defects are also found. They are especially visible with scanning at low bias (blue circles in Fig. S4b) and are ascribed to bulk defects or impurities, for instance inclusion of argon, which was implanted by sputtering at high temperatures, or oxygen.[S14]

S5. Manipulation of surface defects

In Fig. S5 we show a single surface defect imaged with a voltage bias of 50 mV and a current setpoint 150 nA. This procedure resembles many manipulation attempts at very small tip heights (tunneling resistance of about 330 kΩ). With these scanning parameters molecules and adsorbates are typically either displaced on the surface or picked up by the tip.[S18] However, the STM image does not show any indication of displacement of the defect (i.e. no half-moon features or streaking in the image indicative of sudden jumps). Therefore, we conclude that they are atomic defects embedded in the first silver layer.

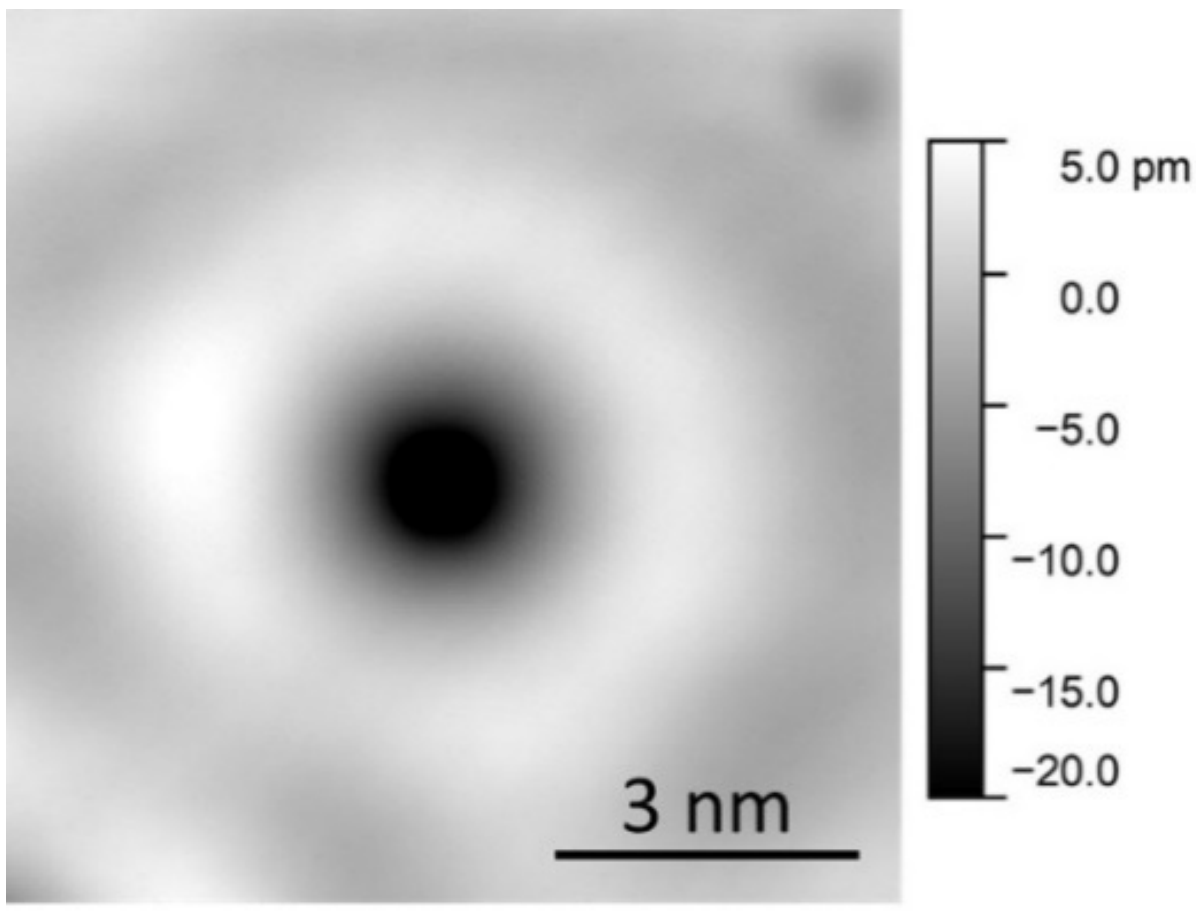

Figure S5. *STM image of a surface defect (150 nA, 50 mV).*

S6. Simulated STM images of surface defects

The surface defects show a bias-dependent appearance – either as a sombrero-hat shape at -2 V or a depression at +2 V (Fig. 3a). Such defects have been assigned to be either S or C atoms.[S17] To identify their chemical nature, we have performed DFT calculations for single sulfur and carbon atoms embedded in the first layer of a Ag(111) surface, and as well as oxygen – a common contaminant. From these calculations STM images were simulated with the Hive Software[S9-S10] using the Tersoff-Hamann approximation[S8] at the same energy as experimental STM images. These simulated STM images show a clear result: while the appearance of sulfur atoms (Fig. S6b) is in very good agreement with the experimental observation (a), the carbon (c) and oxygen (d) atoms at -2 V leads appear as depressions and completely differ from the sombrero-hat shape observed in the experimental STM images. Hence, we conclude that the defects on the Ag(111) surface are single sulfur atoms embedded in the first layer of the silver crystal.

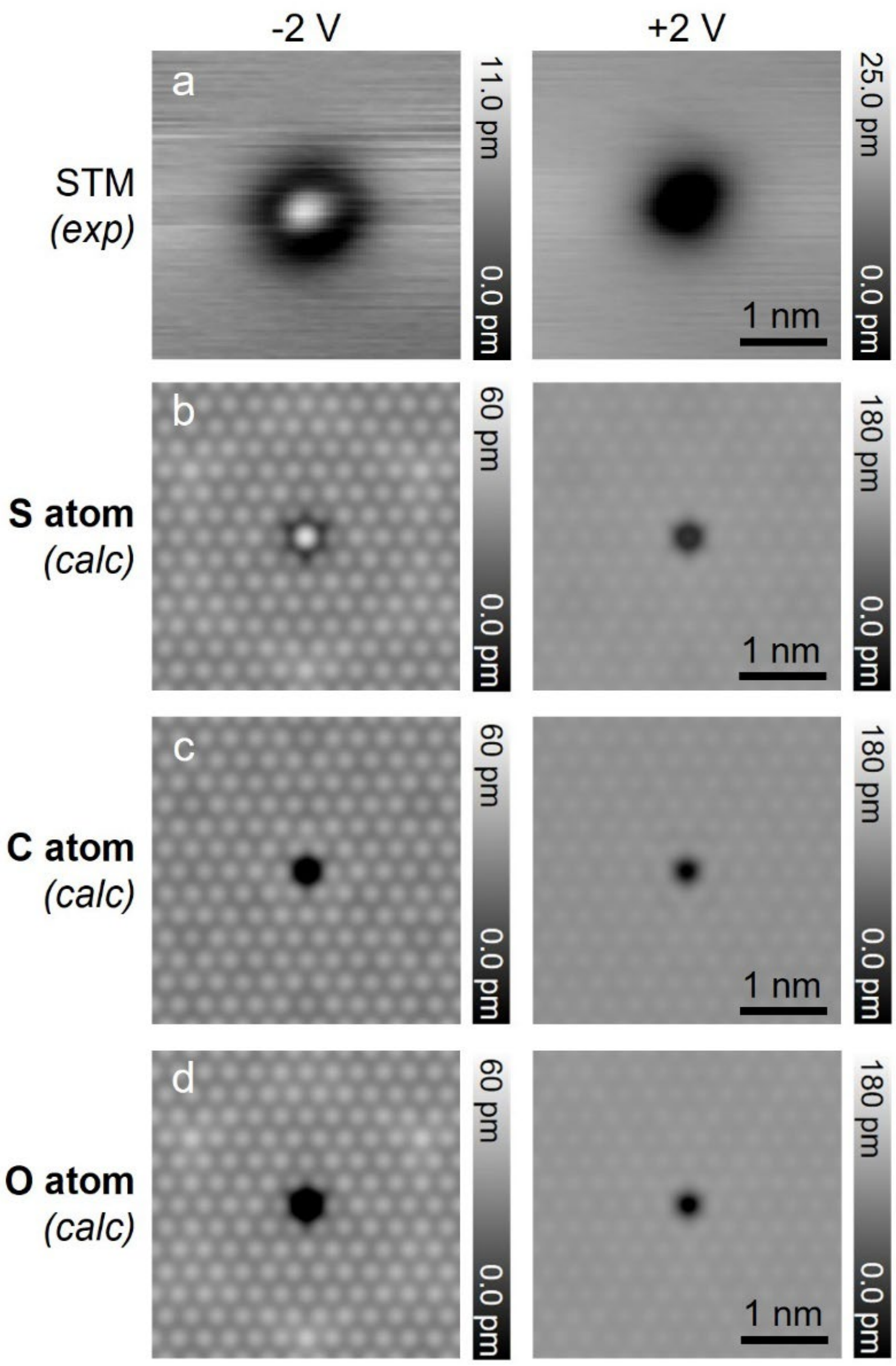

Figure S6. *(a) Experimental STM images of a surface defect recorded at -2.0 V and +2.0 V. (b-d) Simulated STM images of a S atom (b), C atom (c) and O atom (d), substituting an Ag atom of the top layer of a 9 × 9 × 5 slab, respectively. The simulations are obtained through the Tersoff-Hamann approximation using states between -2.0 or +2.0 eV and Fermi, with a constant charge density level of* $2.0 \cdot 10^{-2}$ $e^{-} \cdot Å^{-3}$.

S7. Washboard potential

Figure S7 presents a scheme of the potential energy, experienced by the DBTF molecule in its mobile configuration, along the direction of motion. It consists of two contributions: (1) the shallow surface corrugation, which is the reason for the high molecular mobility in this configuration, with a 2.89 Å periodicity from the Ag(111) lattice; (2) a broad potential well that is caused by the electric field in the STM junction. Due to the tip size, the width of the well is much larger than the surface lattice constant, resulting in a so-called washboard potential. We assumed an idealized STM junction with a charged sphere, representing the STM tip, at a small distance from a metallic surface. The vertical component of the electric field, calculated in each position on the surface, was used to determine the potential energy that is experienced by the DBTF molecule with a vertical electric dipole $\vec{p}_{mol}$ (see Fig. 3d).

Such a washboard potential leads to very different effective diffusion barrier heights, depending on the location of the molecule with respect to the STM tip. If the molecule is rather far away from the tip (*i.e.* at the left/right edge in Fig. S7), the diffusion barrier is essentially given by the surface corrugation and the potential energy curve is roughly sinusoidal with no or a very slight superimposed slope. In contrast, the slope dominates over the sinusoidal modulation if the molecule gets closer to the tip (*i.e.* at a lateral position of about ±10 nm), and the effective energy barrier is therefore strongly reduced (compare the upper inset with the lower inset of the diagram). Consequently, the motion in this region may occur on a shorter timescale than in the surrounding regions[S19] and might therefore contribute less to the total transfer time.

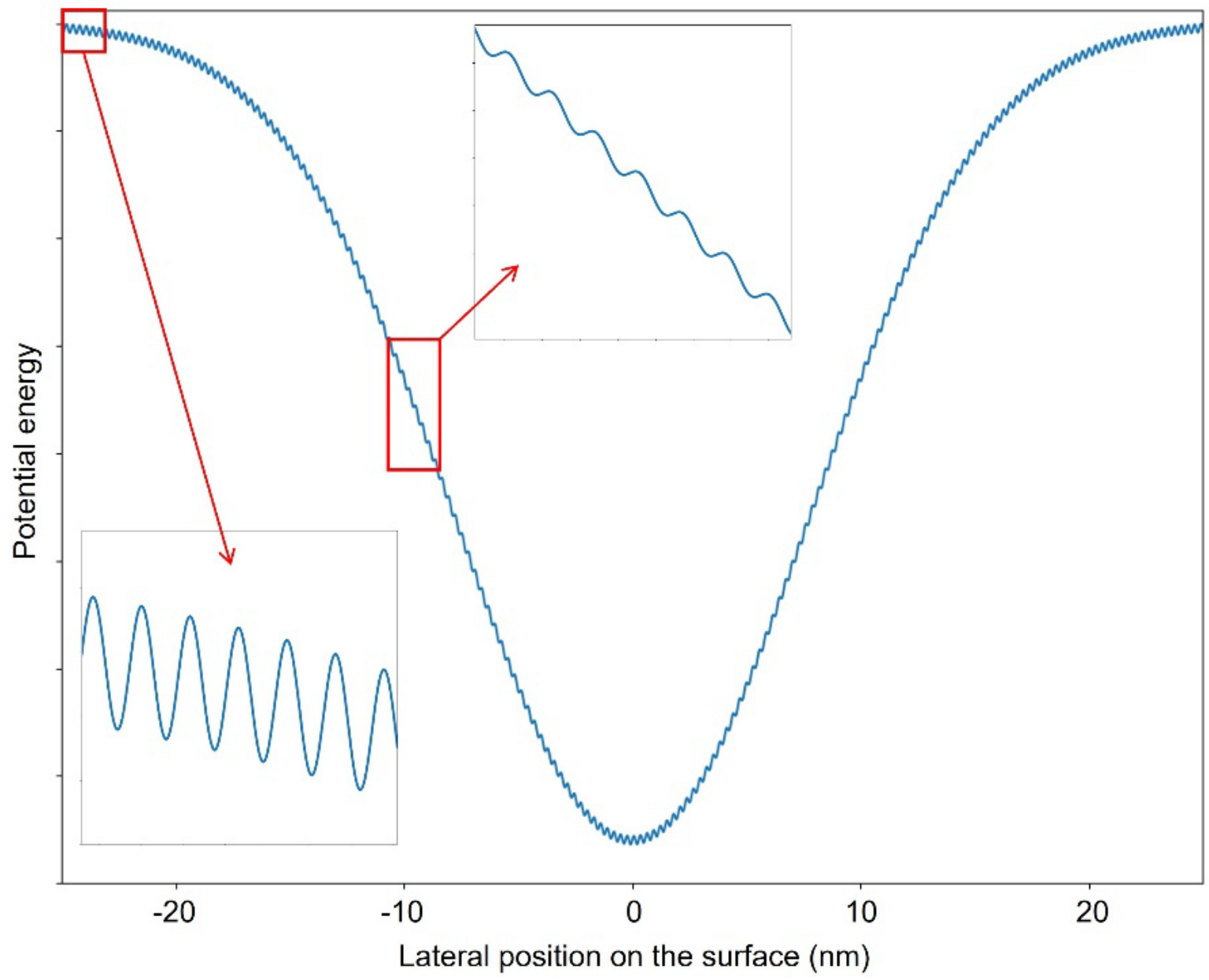

Figure S7. *Scheme of the potential energy as a function of the lateral position (the tip is located at the center of the x-axis) on the surface. The combination of the shallow surface corrugation and the comparably broad potential well caused by the electric field in the STM junction leads to the characteristic washboard potential.*[S20]

S8. Calculated atomic displacements from a sulfur atom defect

We calculated the local distortion of the Ag(111) surface lattice caused by a S atom. Local distortion of the surface lattice might change the potential energy surface and thus the molecular motion. Such an effect has been described theoretically for adatom diffusion.[S21] Experimentally, it is known that the arrangement of surface atoms affects the energetics of adsorbed molecules, *e.g.* adsorption on fcc and hcp domains on Au(111).[S22]

To calculate the relaxed structure for a sulfur atom defect in a Ag(111) surface, a $9 \times 9 \times 5$ slab model (consisting of 404 Ag atoms and 1 S atom) was used. In this model one of the Ag atoms in the top surface layer (layer 4 in Fig. S8a and Fig. S8b) is replaced with an S atom. The top three layers of the slab are then allowed to relax, while the bottom two layers are frozen at the theoretically optimized Ag lattice constant of 4.0742 Å. The in-plane and out-of-plane displacements were then obtained by comparison of the resulting atomic positions with those in a slab consisting of only Ag atoms.

We found that these atomic displacements are quite minor, with the maximum displacement of the Ag-atoms (2.3 pm) being only about 0.6% of the theoretically optimized Ag lattice constant.

The greatest in-plane displacements (Fig. S8a) occur in the top layer of the slab. Here the Ag atoms which are directly adjacent to the embedded S atom (at a distance of 2.88 Å from the S atom) are displaced away from the S atom by 2.3 pm, while the displacement drops below 1.0 pm for Ag atoms at a distance of 8.64 Å from the embedded S atom. Additionally, as shown in Fig. S8b, the maximum out-of-plane displacement for an Ag atom in the top layer of the slab ranges between +1.3 pm and -0.68 pm.

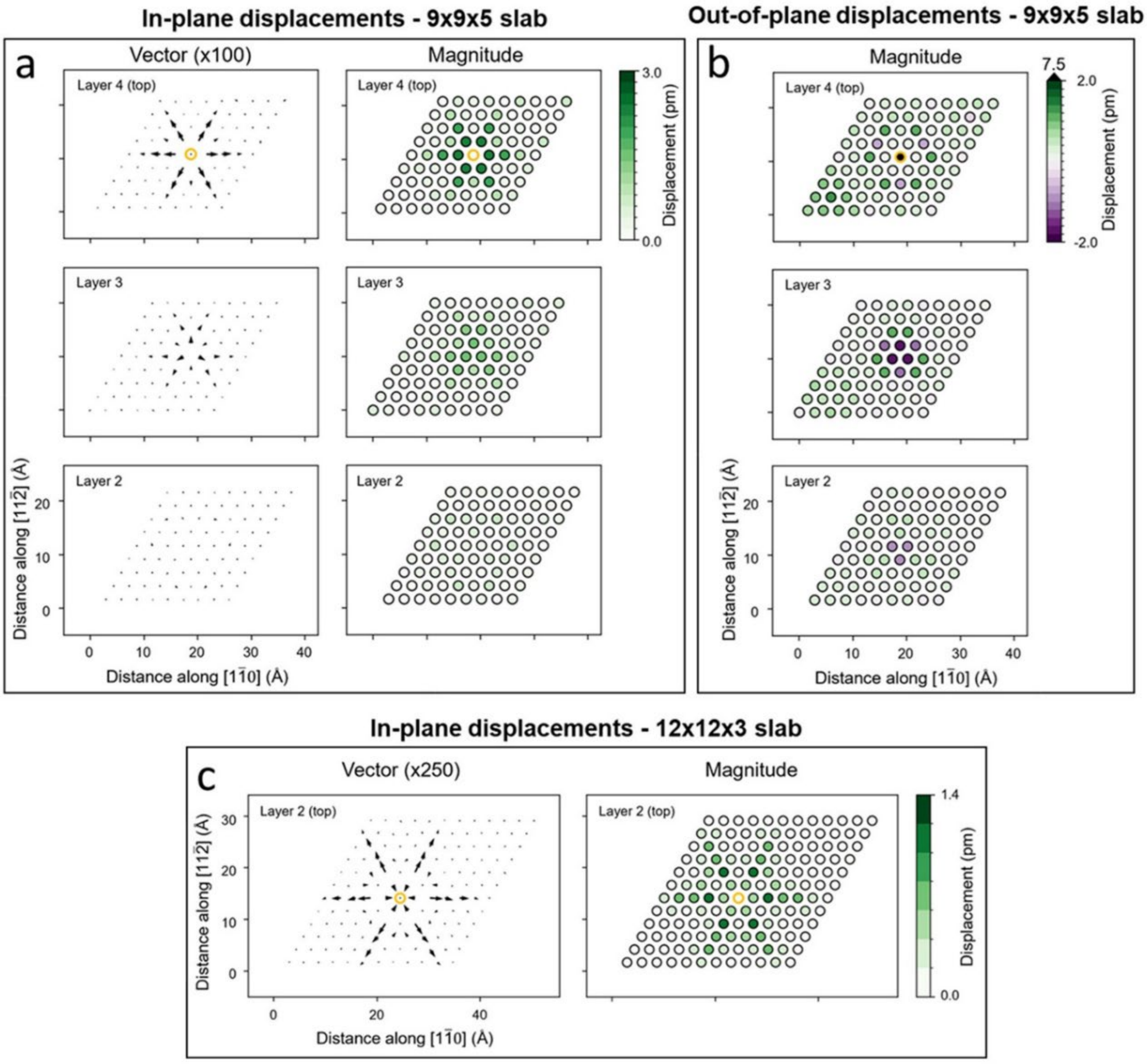

Figure S8. *(a-b) A single sulfur atom (orange circle) is embedded in the top layer (layer 4) of a 9 × 9 × 5 slab of Ag atoms, and the calculated atomic displacements relative to a pristine Ag slab are shown. (a) Top-down, layer-by-layer, view of the in-plane displacements. The left-hand plots show the vectors (multiplied by a factor of 100), while the right-hand plots display the magnitude, with the position of the black circles corresponding to those of Ag atoms in a pristine Ag slab. (b) Top-down, layer-by-layer, view of the out-of-plane displacements. The black circles correspond to the positions of an Ag atom in a pristine Ag slab, while the colour within the circles denotes the magnitude of the out-of-plane displacement. (c) A single sulfur atom (orange circle) is embedded in the top layer (layer 2) of a 12 × 12 × 3 slab of Ag atoms and a top-down view of calculated in-plane atomic displacements relative to a pristine Ag slab is shown. The left-hand plot shows the vectors corresponding to these displacements (multiplied by a factor of 250), while the right-hand plots display their magnitude with the position of the black circles corresponding to those of Ag atoms in a pristine Ag slab.*

To check whether these small displacements were an artifact of the relatively small super cell, as due to the periodic boundary conditions of the calculation there is only 9 unit cells between each S atom, the relaxed structure of a 12 × 12 × 3 slab model (consisting of 431 Ag atoms and 1 S atom) was also examined (Fig. S8c). In this model only the atoms in the top layer were allowed to relax, while the bottom two layers were frozen at the theoretically optimized Ag lattice constant. Again, the atomic displacements are quite minor. As can be seen in Fig. S8c no Ag atom is displaced in plane by more than 1.2 pm, and at a distance of 8.64 Å from the embedded S atom the displacement is only 0.8 pm compared to a 'pristine' Ag slab. This leads us to believe that a sulfur atom defect in a real crystal can only cause a very minor lattice distortion.

Thus, this effect is not only very small in magnitude, but also very local – only those silver atoms within 7.5 Å (*i.e.* three lattice sites) from the defect show a deviation in their lattice constant. This contrasts with our experiments, where the effect of reduced molecular velocity is also present if the molecular pathway is 2 nm away from the sulfur. Therefore, lattice distortions are probably not responsible for the reduced molecular velocity.

S9. Examples of current traces

In Fig. S9 we show different examples of current traces recorded with the STM tip at constant position above a mobile DBTF molecule while the applied bias is a square wave oscillating between +1 and -1 V with different frequencies. The three different examples show that the back-and-forth motion of the molecule can be precisely controlled over a wide range of frequencies (from 10 Hz to 20 kHz) and sustained for many cycles.

In Fig. S9a we show a portion from a current trace where the molecule was attracted toward and repelled from the STM tunneling junction, due to the bias being oscillated at a frequency of 200 Hz. This trace was recorded over several seconds (corresponding to thousands of periods), showing back-on-forth motion of the molecule over the entire duration. When the bias is positive the current is positive with an average value around 3 nA – indicating that the molecule is under the tip. When the bias is negative, however, the current is negative with an average value around -0.5 nA – indicating that the molecule is away from the tip. In Fig. S9b we show 6 seconds of a current trace recorded with the bias oscillating at 10 Hz. As in (a), this current trace shows that at every cycle the molecule moves away from and back beneath the STM tip. Note that, at every bias switch, we observe an inductive spike. In some of the cycles shown in (a), when the bias switches to the negative value, after the inductive spike the current does not drop rapidly to the low current value, but instead remains at a value of -4.5 nA for a fraction of the cycle. This behavior is due to a 'delayed departure' of the DBTF molecule, as described in Fig. S9. Additionally, in (b) the inductive spike is sometimes not visible, which is the effect of low sampling rate, chosen in this case to decrease the file size.

In Fig. S9c we show 1 ms of a current trace recorded with the bias oscillating at 20 kHz. Under these conditions, the oscillation period is very short and the inductive spike resulting from the bias switch (see Fig. S1) becomes prominent. This leads to some difficulties in recognizing the value at which the current stabilizes; however, we can still see that at positive bias the current approaches a value around 17 nA – indicating the presence of the molecule under the tip. On the other hand, at negative bias, i.e. negative current, we can observe that in some cycles the current drops to approximately -3 nA (red line in Fig. S9c), while in others the value of the current approaches -17 nA (blue line). In the first case, the low magnitude of the tunneling

current at negative bias indicates the absence of the molecule under the tip. In the second case, when the magnitude of the tunneling current at negative bias is large (similar to the magnitude of the tunneling current at positive bias), the molecule still remains under the tip. This behavior arises because the time elapsed at negative bias in each cycle (25 µs) is barely sufficient to allow the departure of the molecule, and so in some cases the molecule remains under the tip. The delayed departure of the molecule, and how to control it, is described in Fig. S9.

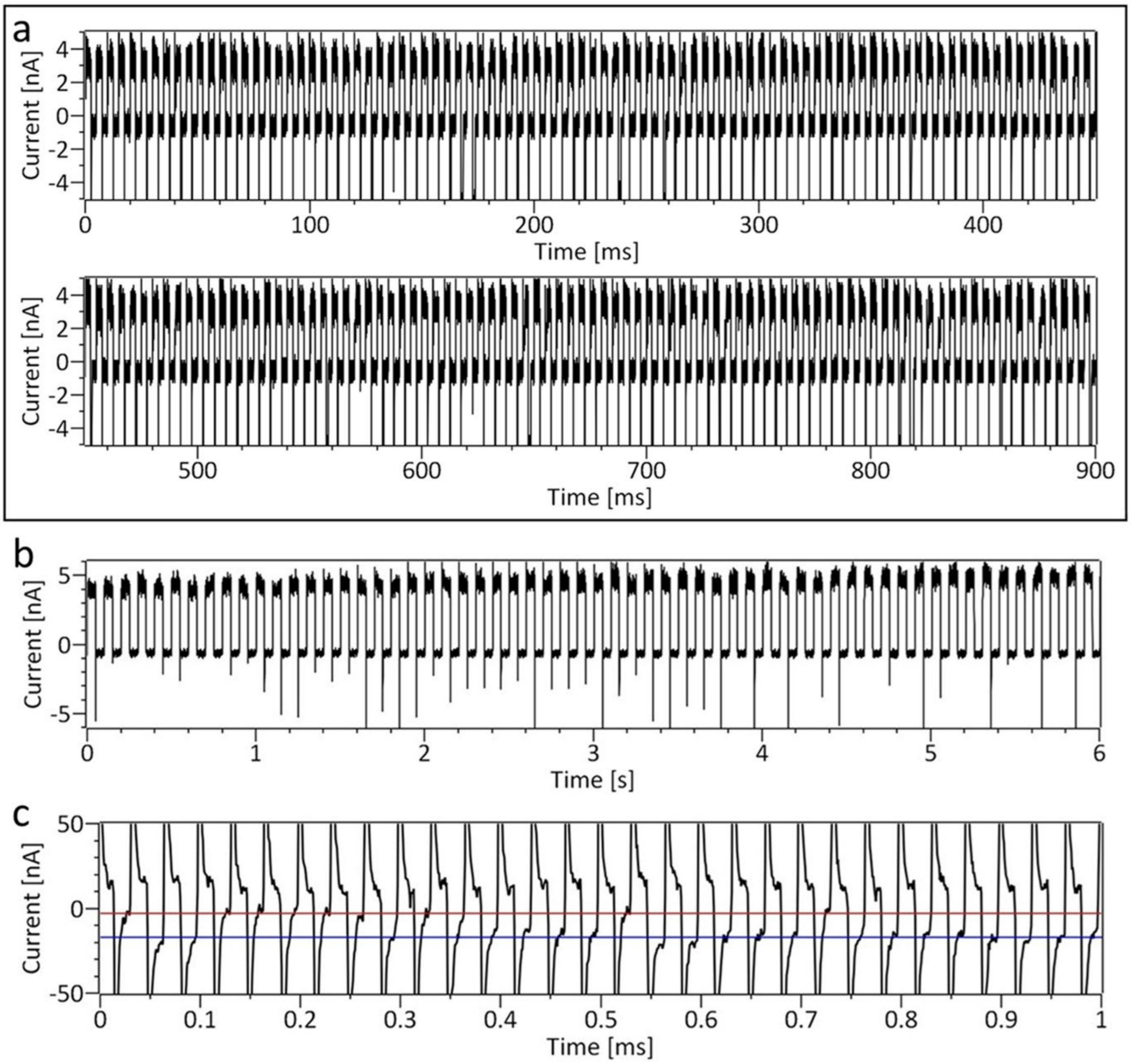

Figure S9. *Current traces recorded with the STM tip at constant position above a DBTF molecule in the mobile orientation, while a square wave of bias oscillating between +1 and -1 V is applied. In panel (a) the bias oscillates at 200 Hz, in (b) at 10 Hz and in (c) at 20 kHz. The red and blue lines in (c) serve as visual guides to indicate the two different values that the current approaches during the negative portion of the bias cycle.*

S10. Delayed departure

We additionally tested the effect of tip height on the controlled motion with oscillating bias voltage. Specifically, we analyzed how the departure of the molecule is affected when the bias is switched from positive to negative. In Fig. S10a we show two current traces recorded with the STM tip at the same position above a mobile DBTF molecule, but at two different heights. The applied bias is a square wave oscillating between +1 and -1.5 V at 10 Hz. The larger negative value in this case (as compared to other experiments) is used to have a higher repulsive interaction – although this does not affect the conclusions, since we are comparing, under the same conditions, the current trace at two different tip heights.

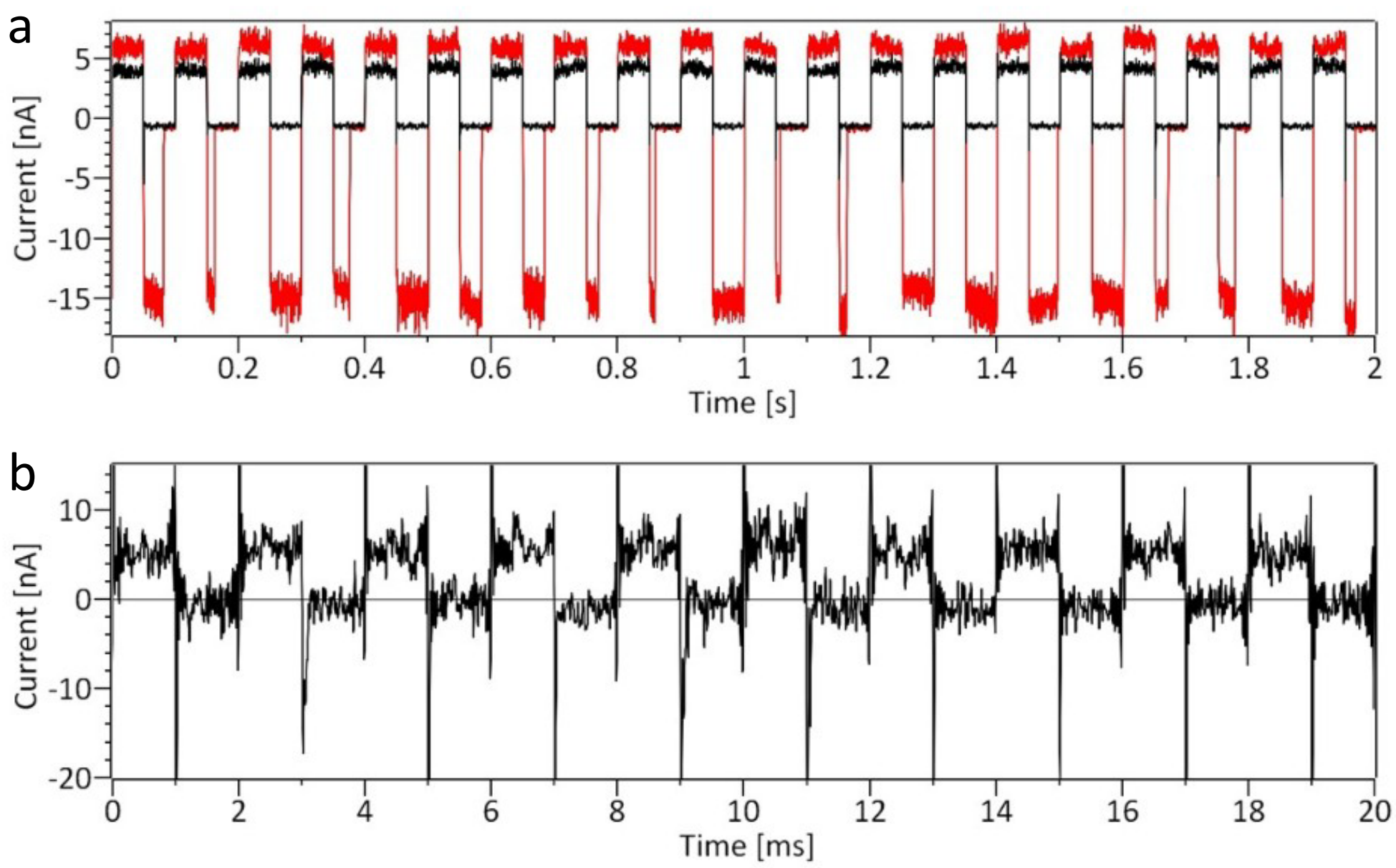

Figure S10. *Current traces recorded with the STM tip at constant position above a DBTF molecule in the mobile orientation while a square wave of bias oscillating between +1 and -1.5 V is applied. The black traces in (a) and (b) are recorded under the same conditions, but with bias frequencies of 10 Hz and 500 Hz, respectively. The red trace in (a) is recorded with the STM tip about 30 pm closer to the sample than for the black trace in (a).*

In the black trace, after the bias switches to the negative value, the current drops directly to approximately -0.5 nA. This indicates the absence of the molecule under the tip with no observable delay. On the other hand, the red trace is recorded in the same conditions as the black one, but with the STM tip about 30 pm closer to the molecule. Under these conditions we observe a clear delay of the departure of the molecule as the current does not drop suddenly to -0.5 nA, but reaches first a value of -15 nA and holds this value for an average time of 30 ± 20 ms. This delay indicates that the molecule remains under the tip for some time at the repulsive bias, likely due to the increased effect of the van der Waals forces.

It should be noted that this experiment was performed with the molecule far from any defects in the surrounding area. Under the same experimental conditions as those described for the black trace, we measured the velocity of the free molecule to be about 570 µm/s (see Fig. 2b). As the molecule is 2.7 nm long from Br to Br and we consider that, before repulsion, the tip is

positioned about the midpoint of the molecule, we calculate that it should take about $\frac{2.7}{2}\frac{1}{570} = 2.4\ \mu s$ for the molecule to move out of the tunneling junction. To resolve this very first instant after the bias switch, under the same conditions as those described for the black trace, we increased the bias oscillation frequency to 500 Hz and obtained the current trace shown in Fig. S10b. The resulting current trace shows indeed only the initial inductive spikes at the bias switch from +1 V to -1.5 V, and the current that drops directly to -0.5 nA with no delay. This confirms that the molecule moves away within the first 5 μs of the spike at the beginning of the bias switch, demonstrating a substantially free molecule which is not experiencing van der Waals trapping.

S11. Alternating bias voltage at different distances from the defect

As described in the main text for the molecule moving between stoppers (*i.e.* S atoms), the return times in regime (1) were too short to be measured, since the free path from the tip position to the defect was very short (14 nm). To use the alternating bias voltages experiments and analyze the return times of the molecule in regime (1), longer return times are needed. This was achieved by extending the pathway along which the molecule could travel before encountering a defect in the following manner: first the molecule was positioned on a close-packed row, at a parallel distance of 0.93 nm to a surface defect, *i.e.* 4 atomic rows (black open circle in Fig. S11a), as in the experiment described in Fig. 4d. Next, the STM tip was positioned at various points along the pathway of the molecule (marked by black circles in Fig. S11a) and the same oscillating bias voltage experiments were performed as those described for Fig. 4 in the main text (note the absence of stoppers in this case).

While performing the alternating bias voltage experiments at 100 Hz, we observed the molecule moving alternately in regime (1) and (2), like the case described in the main text. This observation supports the hypothesis that in this experiment we are in the same conditions as in Fig. 4d and the molecule moves toward that defect. Indeed, at the beginning of the measurement day, the molecule was one atomic row further away from the defect (*i.e.* 5 rows distant) and we observed only one regime. Only after the molecule has moved one row closer to the defect (same distance of the experiment of Fig. 4d-f with the stoppers), did we start to see the two regimes.

Regarding the direction of motion of the molecule it is important to note that if the molecule were to travel in the other direction, that is, toward the step edge (see Fig. S11a), the molecule would become stuck at the step edge instead of returning. We never observed molecules spontaneously detaching from a step edge when the tip was remote from the molecule. In fact, removing a molecule from the step edge the requires the tip to be scanned directly over it. Moreover, if the molecule would travel in some cycles toward one direction and in some cycles toward the other direction, we would observe a bimodal distribution of return times. This would reflect two distinct characteristic return times associated with the return from the two directions. However, as we describe in the following, we observe a unimodal distribution of return times.

Here we analyze only the motion in regime (1) (motion in regime (2) is analyzed in the main text). Since in this experiment the distance between tip and defect is larger, we are able to measure the return times at each cycle, over about 200 cycles.

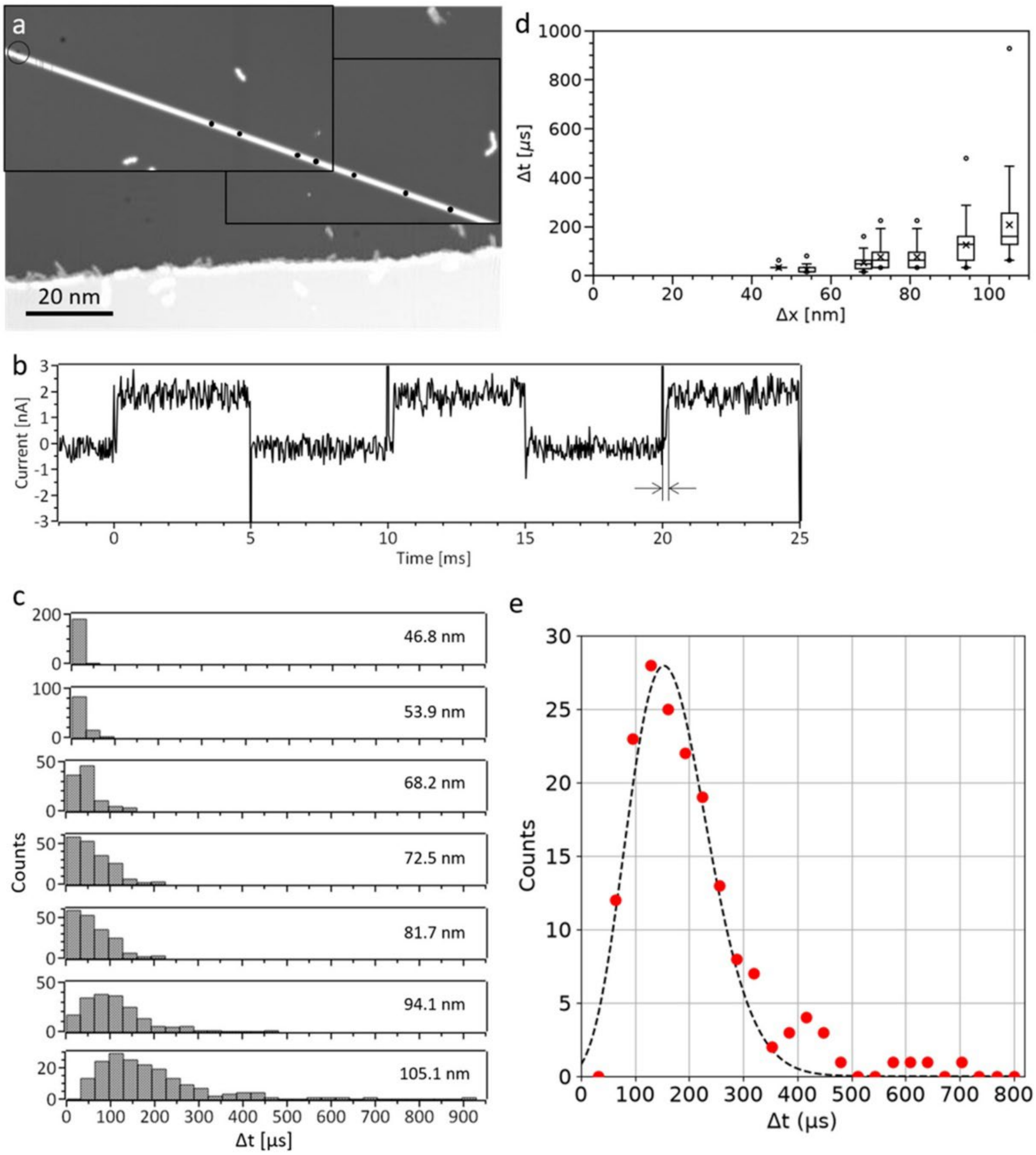

Figure S11. *(a) STM images correctly overlapped of a molecule in the mobile orientation that moves past a nearby surface defect (0.5 V, 56 pA). An open black circle indicates the defect, at 0.93 ± 0.05 nm to the molecular stripe. Black dots at various positions along the stripe show locations at which the tip was held during the oscillating bias measurements. At the bottom of the image a step edge and the upper terrace are visible. (b) Three cycles of the current trace for the tip positioned at 105 nm distance from the defect: the bias switches from -1 V to +1 V at 0 ms, 10 ms, and 20 ms. (c) Histograms (bin width: 32 µs) of measured return times for the tip located at the distances from the defect as indicated, corresponding to the black dots in (a). (d) Box plot of the distributions shown in (c) presented in the Δt-vs-Δx plot. Inside the box there is the 50 % of the data, the line inside the box indicates the median, the cross indicates the mean and the open black dots indicate the minimum and maximum values of the distribution. (e) Fit of the data in (c) at 105.1 nm (plotted as red circles) with a Poisson distribution.*

In Fig. S11b we show an example of three cycles of the current trace, measured with the tip placed on the stripe at a distance of 105 nm from the defect. The return time is indicated with arrows. The histogram of the measured return times for each tip-defect separation is shown in

Fig. S11c. In Fig. S11d we show a box plot of the distribution of the return time values at each tip-defect distance. From this analysis we observe that:

1) The return times appear to follow a Poisson distribution (Fig. S11e). This fits the model of a molecule pulled away in a stochastic manner from the potential well created by the defect.

2) The distributions become broader as the tip is further away from the defect. This observation shows a dependence on the tip-defect distance due to the inhomogeneity of the electric field in the tunneling junction. If this wouldn't be the case, the distributions would be the same at each distance.

3) The median return time is in the order of tens of microseconds, in contrast to larger median delay times observed in the other experiments shown in this work.

4) As the tip is positioned further away from the defect, longer travelling times are expected. However, this dependence on distance is not relevant in the present context, since the stochastic waiting time at the defect masks the underlying time–distance relationship, which is just slightly visible.

To explain this observation, we make the following model. In this setting, i.e. defect at 4 atomic rows (0.93 nm) from the path (experiment of Fig. 4d-f and Fig. S11), we observe two regimes:

- (1) The molecule reaches the defect only to a "weakly locking" position, which is actually visible in Fig. 4d. Note that this "weakly locking" position is observed only if a defect is at 4 atomic rows (0.93 nm) from the path, not for larger separation. This explains the Poissonian distribution of return times, and a median return time in the order of tens of microseconds, since the "weakly locking" position is characterized by a shallow potential well.
- (2) The molecule reaches the "strongly locking" position or goes over it. In this case, the molecule does not come back at each oscillation, because the median return time is larger than the half period of oscillation, since the "strongly locking" position is characterized by a deeper potential well. This condition is observed also for the experiments of Fig. S11, but analyzed in details in the main text, Fig. 4d-f.

References

[S1] L. Lafferentz, F. Ample, H. Yu, S. Hecht, C. Joachim, L. Grill, *Science* **2009**, *323*, 1193-1197.
[S2] G. Kresse, *J. Non-Cryst. Solids* **1995**, *192-193*, 222-229.
[S3] G. Kresse, J. Furthmüller, *Phys. Rev. B* **1996**, *54*, 11169-11186.
[S4] P. E. Blöchl, *Phys. Rev. B* **1994**, *50*, 17953-17979.
[S5] G. Kresse, D. Joubert, *Phys. Rev. B* **1999**, *59*, 1758-1775.
[S6] J. P. Perdew, K. Burke, M. Ernzerhof, *Phys. Rev. Lett.* **1996**, *77*, 3865-3868.
[S7] S. Grimme, J. Antony, S. Ehrlich, H. Krieg, *J. Chem. Phys.* **2010**, *132*, 154104.
[S8] J. Tersoff, D. R. Hamann, *Phys. Rev. B* **1985**, *31*, 805.
[S9] D. E. P. Vanpoucke, G. Brocks, *Phys. Rev. B* **2008**, *77*, 241308(R).
[S10] D. E. P. Vanpoucke, *PhD thesis, University of Twente, The Netherlands* **2009**.
[S11] E. Sanville, S. D. Kenny, R. Smith, G. Henkelman, *J. Comput. Chem.* **2007**, *28*, 899-908.
[S12] G. Henkelman, A. Arnaldsson, H. Jónsson, *Comput. Mater. Sci.* **2006**, *36*, 354-360.
[S13] M. Yu, D. R. Trinkle, *J. Chem. Phys.* **2011**, *134*, 64111.
[S14] C. Sprodowski, K. Morgenstern, *Phys. Rev. B* **2010**, *82*, 165444.

[S15] J. Heintz, C. Durand, H. Tang, R. Coratger, *Phys. Chem. Chem. Phys.* **2020**, *22*, 3173-3183.
[S16] K. Morgenstern, K.-H. Rieder, G. Fiete, *Phys. Rev. B* **2005**, *71*, 155413.
[S17] K. F. Braun, *PhD thesis (FU Berlin)* **2000**.
[S18] S.-W. Hla, K.-F. Braun, K.-H. Rieder, *Phys. Rev. B* **2003**, *67*, 201402.
[S19] M. Schunack, T. R. Linderoth, F. Rosei, E. Laegsgaard, I. Stensgaard, F. Besenbacher, *Phys. Rev. Lett.* **2002**, *88*, 156102.
[S20] J. A. Stroscio, D. M. Eigler, *Science* **1991**, *254*, 1319.
[S21] T. Ala-Nissila, S. C. Ying, *Prog. Surf. Sci.* **1992**, *39*, 227-323.
[S22] M. Vladimirova, M. Stengel, A. D. Vita, A. Baldereschi, M. Böhringer, K. Morgenstern, R. Berndt, W.-D. Schneider, *Europhys. Lett.* **2001**, *56*, 254-260.
